# Diet and Economic Modelling to Improve the Quality and Affordability of the Australian Diet for Low and Medium Socioeconomic Households

**DOI:** 10.3390/ijerph18115771

**Published:** 2021-05-27

**Authors:** Michelle Blumfield, Carlene Starck, Tim Keighley, Peter Petocz, Anna Roesler, Kylie Abbott, Tim Cassettari, Skye Marshall, Flavia Fayet-Moore

**Affiliations:** 1 Department of Science, Nutrition Research Australia, Sydney, NSW 2000, Australia; michelle@nraus.com (M.B.); tim.keighley@gmail.com (T.K.); Peter.petocz@mq.edu.au (P.P.); anna.roesler@flinders.edu.au (A.R.); skye@nraus.com (S.M.); 2 Department of Translational Science, Nutrition Research Australia, Sydney, NSW 2000, Australia; carlene@nraus.com (C.S.); tim@nraus.com (T.C.); 3 Riddet Institute, Massey University, Palmerston North 4474, New Zealand; 4 Nutrition Research Australia, Sydney, NSW 2000, Australia; kylie@nraus.com; 5 Bond University Nutrition and Dietetics Research Group, Faculty of Health Sciences and Medicine, Bond University, Gold Coast, QLD 4226, Australia

**Keywords:** diet, cost analysis, affordability, food security, diet quality, food-processing, socioeconomic

## Abstract

Food costs are a barrier to healthier diet selections, particularly for low socioeconomic households who regularly choose processed foods containing refined grains, added sugars, and added fats. In this study, the objectives were to: (i) identify the nutrient density-to-cost ratio of Australian foods; (ii) model the impact of substituting foods with lower nutrient density-to-cost ratio with those with the highest nutrient density-to-cost ratio for diet quality and affordability in low and medium socioeconomic households; and (iii) evaluate food processing levels. Foods were categorized, coded for processing level, analysed for nutrient density and cost, and ranked by nutrient density-to-cost ratio. The top quartile of nutrient dense, low-cost foods included 54% unprocessed (vegetables and reduced fat dairy), 33% ultra-processed (fortified wholegrain bread and breakfast cereals <20 g sugars/100 g), and 13% processed (fruit juice and canned legumes). Using substitution modelling, diet quality improved by 52% for adults and 71% for children across all households, while diet affordability improved by 25% and 27% for low and medium socioeconomic households, respectively. The results indicate that the quality and affordability of the Australian diet can be improved when nutritious, low-cost foods are selected. Processing levels in the healthier modelled diets suggest that some ultra-processed foods may provide a beneficial source of nutrition when consumed within national food group recommendations.

## 1. Introduction

The habitual diets of Australians are characterized by an inadequate intake of the recommended core food groups and overconsumption of discretionary foods (those not necessary to provide essential nutrients), leading to insufficient intakes of dietary fiber, vitamin A, vitamin D, folate, calcium, and iron, as well as excessive intakes of added sugars, saturated and trans fats, and sodium [1,2,3]. Processed and ultra-processed foods represent over 40% of foods consumed by Australians [4], and have been suggested to contribute to unhealthy dietary patterns and the subsequent increased risk of non-communicable diseases (e.g., type 2 diabetes and cardiovascular disease) and mental illness [4,5,6]. Therefore, the diet quality of the Australian population remains a public health priority [7,8].

A key barrier to equal access of healthy diets is cost, which is particularly relevant in the 2020–2021 Australian recessionary environment [9]. The International Network for Food and Obesity/NCDs Research, Monitoring and Action Support (INFORMAS) protocol provides a framework for examining the price differential of healthy and unhealthy diets [10,11]. Dietary modelling using the INFORMAS protocol has shown that a healthy diet can cost less than the current Australian diet, across a range of household types and socioeconomic status (SES) positions [12]. However, the healthy diet is lower in energy, and dietary modelling in New Zealand suggests that when diets are matched for energy a healthy diet becomes more expensive [13,14]. Food prices vary across Australia, with rural and remote areas showing prices that are up to 40% higher than those in capital cities [7]. The contribution of the food environment to both diet quality and affordability reveals a need for greater understanding about the relationship between nutrition and food cost in Australia, particularly in vulnerable groups.

When food costs are greater than 25% of disposable income, a household is considered to be in ‘food stress’, and when greater than 30% of disposable income, a healthy diet becomes ‘unaffordable’ [9]. Lower and medium SES households in Australia spend a greater proportion of their income on food (15–27% and 12–18%, respectively) as compared with higher SES households (9–13%) [15]. Lower and medium SES households are most affected by regional variations in food prices, inflation, and government policies [7]. Research from the USA [16] and New Zealand [17] have identified a selection of nutrient dense, low-cost foods, including milk, potatoes, breakfast cereals, and eggs [16,17]. However, it is unclear whether similar low-cost foods can be applied in Australia to create a healthier diet, and if these foods positively impact diet quality and affordability for Australian households.

Processed and ultra-processed foods that contain refined grains, added sugars, and added fats have been highlighted as some of the lowest-cost sources of dietary energy, and represent both core (contributing to nutrient requirements) and discretionary (negligible contribution to nutrient requirements) foods [18]. Although ultra-processed foods are linked with poor diet quality and negative health outcomes, a recent study of foods in the USA found that 17% and 33% of ultra-processed and processed foods, respectively, can be classified as nutrient dense [19], and some ultra-processed and processed foods have been classified as both low-cost and nutrient dense (e.g., fortified cereals, beans, milk, and yoghurt) [20,21]. The NOVA processing classification system is the most widely used method for categorizing foods according to their processing level (unprocessed or minimally processed, culinary processed, processed, and ultra-processed) [22]. Australian research has highlighted some disagreements between NOVA classifications and dietary guidelines [23], where some ultra-processed foods are core foods rather than discretionary. Therefore, processing level should be considered when investigating nutrient dense, low-cost foods and their impact on diet quality and affordability in Australia.

To understand this complex relationship between healthy food and affordability, this study aimed to: (i) identify the nutrient density-to-cost ratio of Australian foods, (ii) model the impact of substituting foods with lower nutrient density-to-cost ratio with those with the highest nutrient density-to-cost ratio on diet quality and affordability for low and medium socioeconomic households, and (iii) evaluate food processing level. It is hypothesized that substituting foods in the current diet with foods in the top quartile of nutrient dense, low-cost foods will improve the quality and affordability of modelled diets.

## 2. Materials and Methods

This study was informed by the INFORMAS protocol [11] and reported according to the Strengthening the Reporting of Observational Studies in Epidemiology (STROBE) checklist for cross-sectional studies [24], and Consolidated Health Economic Evaluation Reporting Standards (CHEERS) Statement [25].

### 2.1. Nutrient and Food Price Databases

#### 2.1.1. Australian Food and Nutrient Database

The Australian Food and Nutrient (AUSNUT) 2011–2013 database was developed by Food Standards Australia and New Zealand (FSANZ) to enable food, dietary supplement, and nutrient intake estimates to be made from the 2011‒2013 Australian Health Survey [26]. The AUSNUT database was selected for this study in preference to the current Australian Food Composition Database; therefore, the composition could link directly with dietary intake data, sourced from the Australian 2011–2012 National Nutrition and Physical Activity Survey (NNPAS) [3]. The AUSNUT database contains macro- and micronutrient composition data for 5740 Australian foods, and organizes foods according to a major (2-digit coded, 22 food groups), sub-major (3-digit coded, 132 food groups), and minor (5-digit coded, 515 food groups) food group, with individual foods given an 8-digit code. The 8-digit code forms the basis of a survey ID within the NNPAS [26]. In this study, sub-major (3-digit coded) food groups were aggregated into a reduced number of food categories; a method previously used in dietary modelling, to simplify and combine similar food groups [27]. Mixed dishes, takeaway foods, breads and rolls with flour not defined, organ meats and offal, tea and coffee, water, alcohol, supplements, infant formula, and baby foods were excluded in this study due to the following: being unfeasible to model as individual food categories (e.g., mixed dishes and takeaway foods); contributing negligible calories to the diet (e.g., tea and coffee, water, and supplements); contributing negligible nutrients to the diet (e.g., alcohol); or being consumed in low frequency (e.g., breads and rolls with flour not defined, organ meats and offal, infant formula, baby foods). A total of 57 food categories were analyzed (Table S1).

Within each food category, three representative foods were selected for being the lowest in cost and readily available in major Australian supermarkets, as informed by the INFORMAS protocol [11]. Nutrient data for each food category were based on the average nutrient composition of the three representative foods within each category, obtained from the AUSNUT survey ID (8-digit code). Minor (5-digit coded) food groups were used to classify food categories according to level of fortification (if fortified or not). NOVA processing levels were applied (unprocessed/minimally processed, culinary processed, processed, and ultra-processed).

#### 2.1.2. Food Price Database

An Australian food price database was created according to the INFORMAS protocol [11] and published elsewhere [28]. Food price data were collected from two supermarkets (Coles and Woolworths) each located in a low (Merrylands and Auburn) and medium (Zetland and Burwood) metropolitan SES area within the state of New South Wales, Australia, from 7 December to 11 December 2020. Locations were selected based on the Australian Bureau of Statistics Index of Relative Socioeconomic Advantage and Disadvantage (IRSAD) [29]. The IRSAD summarizes information about the economic and social conditions of people and households within an area by postcode, taking into account both relative disadvantage and advantage [29]. The index ranks areas on a continuum (1 to 10) from most disadvantaged to most advantaged. IRSAD quintiles 1 and 3 were chosen to represent low and medium SES areas, respectively [29].

Price data were collected for each representative food according to the following criteria: (i) the lowest non-discounted price was chosen for the most commonly available product size; (ii) the product was widely available nationally; (iii) fresh produce of poor quality was omitted; and (iv) if a specified product was not available, a similar product or the closest alternative based on its nutrient composition was selected (e.g., a pear if apple unavailable). One sample was collected per representative food product per store, and the average food price per 100 kcal was determined [16,30].

#### 2.1.3. Dietary Intake Database

Dietary intake data were obtained from the NNPAS [3], in order to construct a current diet that accurately represented the Australian population from both low and medium socioeconomic areas. The NNPAS is a nationally representative survey that forms part of the 2011–2013 Australian Health Survey (n = 9341 adults and n = 2812 children) [31]. The automated multiple-pass 24-hour dietary recall method was used to capture all foods and beverages consumed by respondents within the 24 hours prior to the interview day. For children aged 2–14-years, an adult was interviewed on the child’s behalf. This study excluded repeated 24-hour recalls which were performed on a subsample of the participants. Further survey details including sampling methodology and response rates are available in the Australian Health Survey Users’ Guide, 2011–2013 [32].

### 2.2. Categorization of Food Categories by NOVA Processing Level and Discretionary Status

Each food category was coded according to its level of processing using the NOVA classification system [22,33,34] as follows: Group 1, unprocessed or minimally processed foods (e.g., rice, pasta, traditional breads and other cereals, meat, fish, milk, eggs, fruit, roots and tubers, vegetables, nuts, and seeds); Group 2, processed culinary ingredients (e.g., sugar, plant oils, and butter); Group 3, processed foods (e.g., processed breads and cheese, canned foods, and salted and smoked meats); Group 4, ultra-processed foods (e.g., confectionary, savory snacks, fast food dishes, ready-to-eat breakfast cereals, mass-produced packaged breads, frozen and ready meals, and soft drinks). We followed a previously published methodology [4]. Two accredited practicing dietitians (APDs) independently applied the NOVA classification system to each food category. Where a food category contained more than one processing level, the processing level that occurred in the highest proportion of foods within that food category was selected (e.g., legumes and legume dishes were categorized as processed). Any differences in classifications were discussed in the first instance or resolved by a third APD.

Food categories were further coded as non-discretionary or discretionary, according to the Australian Bureau of Statistics discretionary food list [35]. If a food category contained both discretionary and non-discretionary foods, the status occurring in the highest proportion of foods within that food category was selected (e.g., ready-to-eat fortified breakfast cereals with greater than 20 g sugars/100 g were categorized as discretionary, although some (<50%) of the breakfast cereals within that category were not discretionary).

### 2.3. Dietary Modelling Protocol

The dietary modelling protocol is summarized in Table 1.

#### 2.3.1. Step 1: Nutrient Density-to-Cost Ratio of Food Categories and NOVA Processing Levels

The Nutrient Rich Food Index (NRF9.3) per 100 kcal was calculated for each food category [36]. The index assesses the proportion of nutrient requirements that are provided by a food, in relation to energy [16,36]. There are nine positively weighted nutrients (protein, dietary fiber, calcium, iron, potassium, magnesium, and vitamins A, C, and E), and three negatively weighted nutrients (saturated fat, added sugars, and sodium). Food categories with the highest NRF9.3 scores contain higher levels of the positively weighted nutrients and negligible levels of the negatively weighted nutrients. The NRF9.3 was adapted for the Australian Dietary Guidelines and Nutrient Reference Values (Supplementary Methods S1) and a constraint that the ratio could not fall below zero was applied. The nutrient density-to-cost ratio for each food category was calculated as the mean of the ratios for the representative foods chosen in each food category. Food categories in the highest quartile of nutrient density-to-cost ratio were considered to be ‘nutrient dense and low-cost’ foods.

#### 2.3.2. Step 2: Substitution Modelling to Create Low-Cost, Healthier Diets

Current Australian diets for low and medium SES households were constructed using the food categories developed in Step 1. The reference household structure was comprized of four individuals: female, 7 years; male, 14 years; female, 45 year; and male 45 years, as informed by the INFORMAS protocol [11]. The reference household was used for both low and medium SES groups [29]. To maximize the sample size, reference ages were expanded to reflect the age groups in the Australian Nutrient Reference Values as follows: female, 4–8 years; male, 9–13 years; female, 31–50 years; and male, 31–50 years [37]. The demographics of the low and medium SES households modelled in the current study are outlined in Table 2. The most recent equivalized disposable income per week for both low and medium SES households was sourced from the 2017–2018 Australian Survey of Income and Housing [38], which used the Organization for Economic Co-operation and Development (OECD) equivalized disposable income. This is multiplied by an adjustment factor to equivalize to the INFORMAS reference household [11]. For a 4-person household, the OECD adjustment factor is 2. Total household dietary intakes were calculated by summing the daily intakes of all household members and multiplying by 7 to provide dietary data over a week. The cost of each diet was expressed as a percentage of the disposable income for each household per week to reflect affordability.

All substituted healthier diets aimed to meet Australian food group recommendations, with no allowance for discretionary foods [39]. The Australian core food groups were labelled as fruit, vegetables, cereals and grains, dairy and alternatives (milk, yoghurt, cheese, and/or alternatives), meat and alternatives (lean meat, fish, poultry, eggs, tofu, nuts, and legumes), and other foods (margarines and edible oils). An algorithm was created to replace food categories in the current diet with food categories from the highest quartile of nutrient density to cost, as identified in Step 1 of the modelling protocol. Each substitution was made according to the ‘like for like’ principle, whereby each food category in the current diet was substituted for a healthier (more nutrient dense) version of that food, keeping as close to the original food as possible. For example, white bread was substituted with wholegrain bread or processed meat was substituted with lean fresh meat. Discretionary foods were replaced according to the same ‘like for like’ principle, with one discretionary serving equivalent to 150 kcal. The algorithm was checked for logical output using key foods. Full detail regarding the substitution rules is provided in Supplementary Methods S2 and Table S2.

#### 2.3.3. Diet Quality

The diet quality of modelled adult diets was examined using the validated Healthy Eating Index for Australian Adults (HEIFA-2013) [40]. The HEIFA-13 was calculated based on an 11-component system of five food groups (vegetables, fruits, cereals and grains, dairy and dairy alternatives, and meat and meat alternatives), three negative nutrients (fats, added sugars, and sodium), water intake, and alcohol intake. Both current and healthier modelled diets were given the maximum (healthiest) score for Component 7 (water) and Component 11 (alcohol), as these foods were excluded from the current study.

The Dietary Guidelines Index for Children and Adolescents (DGI-CA) was used to assess the diet quality of children’s diets [41,42,43]. The DGI-CA was calculated based on 11 components (5 core food groups; intake of wholegrain bread and reduced-fat dairy foods; intake of extra foods that are nutrient poor and high in fat, salt, and added sugars; healthy fats and oils; water; and diet variety). Both current and healthier modelled diets were given the maximum (healthiest) score for water (Component 6), as this food was excluded from the current study.

### 2.4. Statistical Analyses

Statistical analyses were performed using the R programming language (version 4.0.3, R Core Team, Vienna, Austria) [44], with extensive use of the tidyverse packages (R Studio, Boston, MA, USA) [45]. Mean (SD) was used to describe population, nutrition, and cost input data, and mean (SEM) was used to present dietary data produced from the substitution modelling protocol. The recommended serving size, as determined by the Australian Guide to Healthy Eating [39], was recorded.

Statistical significance, for comparison of nutrition and cost between diets, was obtained from a general linear model with SES, age-sex category, and their interaction as fixed factors. A linear combination of adult male + adult female + child male + child female was used to obtain values for a model ‘household’ of two parents and two children. Values of *p* < 0.005 were taken to represent statistical significance, adjusted down from *p* < 0.05 using a Bonferroni approach for multiple comparisons within groups of variables.

## 3. Results

### 3.1. Nutrient Density, Cost, and the Nutrient Density-to-Cost Ratio of Australian Food Categories

**Figure 1:**
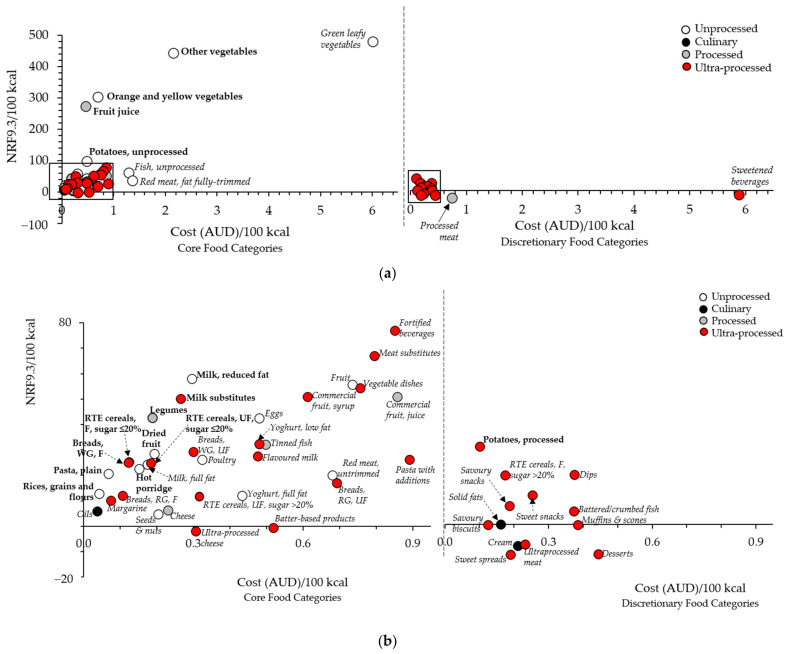
Nutrient density (NRF9.3) per 100 kcal vs. cost (AUD per 100 kcal) for the 57 food categories. (**a**) Full graph illustrating the relationship between nutrient density and cost for all 57 food categories, separated as core or discretionary food categories. The majority of food categories are clustered with an NRF9.3/100 kcal less than 80 and a cost (AUD)/100 kcal) less than AUD 0.9 (boxed areas). The food categories in the highest quartile of the nutrient density-to-cost ratio are shown in bold, food categories in Quartiles 2–4 are in italics; (**b**) enlargement of the clustered food categories contained within the boxes in Figure 1a for each core and discretionary food category. The food categories appearing in the highest quartile of the nutrient density-to-cost ratio are shown in bold, and food categories in Quartiles 2–4 are in italics. Figure abbreviations: F, fortified; RTE, ready to eat; RG, refined grain; UF, unfortified; WG, wholegrain.

The nutrient density of each of the 57 food categories (defined by NRF9.3/100 kcal), was plotted as a function of cost (AUD/100 kcal), as shown in Figure 1a, in which core and discretionary food categories are shown separately on distinct sets of axes. Nutrient densities (per 100 kcal) ranged from −22.3 (processed meat, with cost at AUD 0.74 per 100 kcal) to 479.2 (green leafy vegetables, with cost at AUD 6.01 per 100 kcal). Green leafy vegetables, the highest nutrient density food category, was also the most expensive. The majority of food categories were clustered around a nutrient density score of less than 100/100 kcal and a cost less than AUD 1/100 kcal (Figure 1b). Processing levels were evenly distributed throughout all nutrient density and cost levels for core foods (left-hand axes), but there were no unprocessed food categories in discretionary foods (right-hand axes).

The nutrient density score (NRF9.3/100 kcal) for the top quartile of nutrient dense, low-cost food categories ranged from 12.9 ((SD 4.6) rice, grains and flours) to 443.0 ((SD 160.6) other vegetables); the only discretionary food category included was processed potatoes (e.g., commercial oven potato fries, 37.3 (SD 4.9)) (Table 3). Rice, grains, and flours were the most affordable food category (0.04 [SD: 0.0] AUD/100 kcal), and other vegetables were the most expensive (2.2 (SD 1.4) AUD/100 kcal). Dried fruit had the lowest nutrient density-to-cost ratio within the top quartile of nutrient dense, low-cost food categories (171.0 (SD 80.1)) and fruit juice had the highest (650.1 (SD 318.9)) (Table 3), followed by orange and yellow vegetables, processed potatoes, and rice, grains, and flours. No food categories from ’other foods’ were in the top quartile (Supplementary Table S3. The difference in scores between the top (first) and second nutrient density-to-cost ratio quartiles was small (≤100) for fruit, cereals and grains, and dairy and dairy alternatives (Supplementary Table S3). The top quartile of nutrient density-to-cost ratio contained 54% unprocessed, 13% processed, and 33% ultra-processed food categories.

### 3.2. The Current Australian Diet

All members in each household reported a low intake across all core food groups, except for females aged 4–8 years from medium SES households who achieved the recommended two servings of fruit per day (Table 4). Discretionary servings exceeded the maximum of two servings per day for all participants. All diets were high in saturated fat and sodium, and low in dietary fiber, Vitamin E, and potassium.

Total food costs for medium SES households were AUD 140.90 per week as compared with AUD 129.70 per week for low SES households. Despite having higher total food costs, food was more affordable in the medium as compared with low SES households (Table 4).

### 3.3. The Modelled Healthier, Low-Cost Australian Diet

For both households, intakes of core food groups increased in the healthier diet as compared with the current diet (*p* < 0.001 for all), despite an overall decrease in energy intake (*p* < 0.001), with the exception of fruit which showed no change (Table 5). As expected, due to the substitution modeling rules, discretionary intake was reduced to zero (Rule 1, Supplementary Methods S2). The majority of macronutrient intakes improved for all members of both households. Total fat reduced by approximately 27% (*p* < 0.001), saturated fat fell below the recommendations of less than 10% of energy intake to approximately 3.9% (*p* < 0.001), and polyunsaturated fat intake increased to 12% of energy (*p* < 0.001). Although total sugars increased (*p* < 0.001), added sugars and free sugars both decreased to below 2% of energy (*p* < 0.001). Dietary fiber and vitamin A levels increased (*p* < 0.001) to meet reference values (Table 5 and Supplementary Table S4. However, protein intake decreased in the healthier diet for the medium SES household by 7% (*p* < 0.001).

Concurrent with the overall improvement in nutritional composition of the healthier diets (Table 5) as compared with the current diets (Table 4), for all household members, there was a significant decrease of 25–27% in total food costs for both low and medium SES households (reduced to AUD 97.60 per week and AUD 102.60 per week, respectively, *p* < 0.001), and a concomitant improvement in diet affordability. When matched for energy, the healthier diet remained lower in cost than the current diet for both low and medium socioeconomic households (data not shown).

### 3.4. Diet Quality of the Current and Healthier Modelled Diets

**Figure 2:**
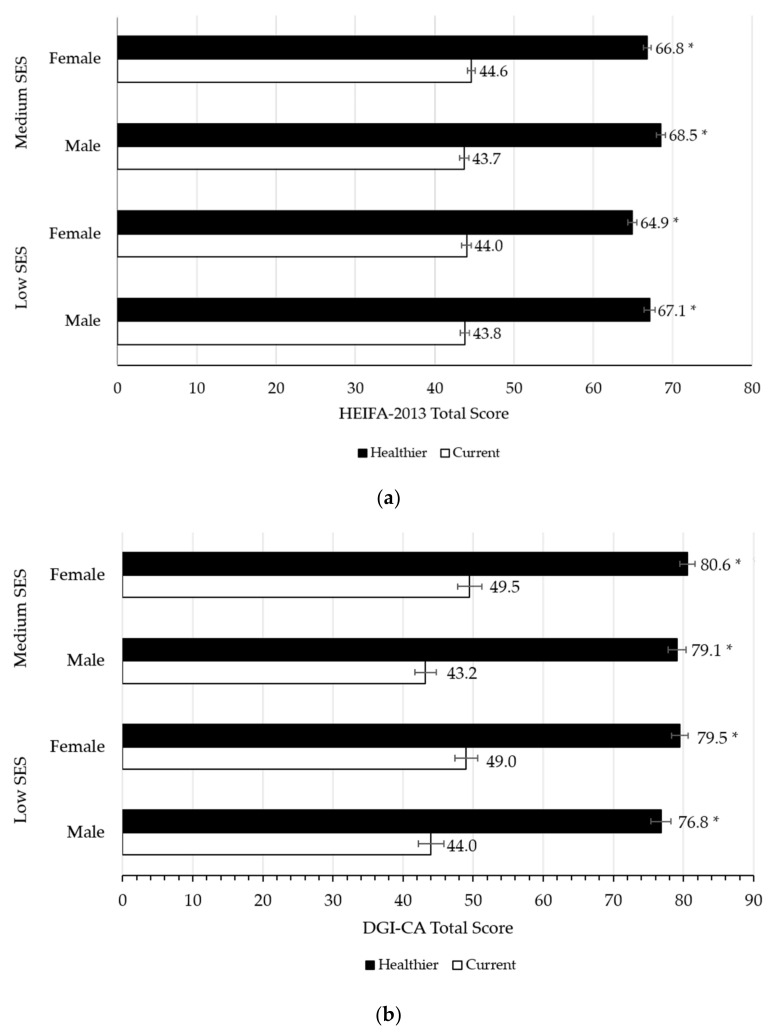
Diet quality of the current and healthier, low-cost diets, using validated diet quality indices. (**a**) HEIFA-2013 (Healthy Eating Index for Australian Adults) [40] assessment of the adult male and adult female current and healthier, low-cost diets, from each of the low and medium SES households; (**b**) DGI-CA (Dietary Guidelines Index for Children and Adolescents) [41,42,43] assessment of the male and female children’s current and healthier, low-cost diets, from each of the low and medium SES households. Statistical significance is denoted by * *p* < 0.005. Comparisons are between current and healthy diets within each group. SES, socioeconomic status.

The healthier, low-cost diet showed an improvement in diet quality over the current diet for all members of both households (adult diets ranged from 64.9 to 68.5 vs. from 43.7 to 44.6 (Figure 2a); children’s diets ranged from 76.8 to 80.6 vs. from 43.2 to 49.5; *p* < 0.001 for all (Figure 2b). Diet quality subscores improved for all members of both households (<0.005 for all), except for the fruit subscore (Supplementary Table S5).

### 3.5. Distribution of NOVA Processing Levels and Food Category Types throughout the Current and Healthier, Low-Cost Diets for Low and Medium SES Households

Current and healthier, low-cost diets contained foods from all NOVA processing categories (Table 6). Ultra-processed foods contributed approximately 60% of the current diet for both households, almost double that of unprocessed foods (approximately 32%), and eight and half times that of processed foods (7%). In the healthier, low-cost diets, unprocessed foods (*p* < 0.001 for all) and processed foods increased (*p* < 0.001 for all), and ultra-processed foods decreased (*p* < 0.001 for all), for both low and medium SES households. The increase in processed foods was attributed to legumes and legume dishes. Processed culinary foods also increased, attributed to unsaturated oils (Table 6). While ultra-processed food categories decreased overall, the remaining 24–25% of ultra-processed food categories represented in the healthier diets were increased from current diets (commercial fruit in syrup, wholegrain breads, ready-to-eat cereals with <20 g sugars/100 g, and dairy milk alternatives).

The healthier, low-cost diets for both low and medium SES households, were higher in unprocessed vegetables (66–67%, from 21%), reduced fat milk (11–12%, from 7%), unsaturated fats and oils (100%, from 13%), legumes and legume dishes (89–90%, from 5%), commercial fruit in syrup (2–3%, from 0.1%), wholegrain breads (53–57%, from 8%), ready-to-eat breakfast cereals with ≤20 g sugars/100 g (15–18%, from 6–8%), and dairy milk alternatives (23–24%, from 0.5%) as compared with the current diet (*p* < 0.001 for all). There was a concomitant decrease (i.e., no inclusion in the healthier diet) in full-fat milk, cheese, whole fruit, refined breads and flour products, ultra-processed milk and milk-based beverages, and animal protein sources (red meat, poultry, eggs and fish) (*p* < 0.001 for all).

## 4. Discussion

This was the first study to investigate the impact of substituting the current diet with foods that are low cost and nutritious, on the diet quality and affordability in Australia, with a secondary focus on food processing level. The findings suggest that the quality and affordability of the Australian diet can be improved concurrently, for both low and middle SES families, and that the low cost and nutritious diet includes foods of all processing levels, supporting hypotheses. In theoretical models, when foods in the Australian diet were replaced with nutrient dense, lower-cost alternatives, diet quality improved by 52% for adults and 71% for children across all households, while diet affordability improved by 25% and 27% for low and medium SES households, respectively. Cost-effective nutrient dense foods were identified across all processing levels, but were predominantly unprocessed (54%, unprocessed potatoes, orange/yellow and other vegetables, and reduced fat dairy) and ultra-processed (33%, fortified wholegrain bread and RTE cereals with less than 20 g sugars/100 g). Top quartile food categories with the highest nutrient density scores included foods from each of the five core food groups (vegetables, fruit, grains, meat/alternatives, and milk/alternatives).

The nutrient density-to-cost ratio was useful as it provided public health guidance for the selection of foods that improved nutrition at the most affordable cost [16,18]. Whilst findings of this study are pertinent for low and medium SES households, the reduction in food costs of 25–27% is also relevant for all SES households during times of economic instability, such as during the COVID-19 pandemic [9]. Although environmental sustainability was not considered in the current study, the results partly align with environmental sustainability-focused dietary modelling in New Zealand, where similarities were seen for changes in milk, potatoes, wholegrain flour, and legumes [17]. There is a need for follow-up research to translate this theoretical finding into practice to demonstrate that it is possible for low and medium SES families to eat a more nutritious diet, and at a lower cost, than their current diets.

The findings confirm that the top quartile of nutrient dense, low-cost food categories were across all core food groups, which suggests that nutrient dense, low-cost food choices are available to meet Australian dietary recommendations [39]. However, a number of nutrient dense food categories, that are culturally important and recommended in dietary guidelines as part of the core food groups, were removed during the modelling because their nutrient density-to-cost ratio did not fall within the top quartile, namely whole fresh fruit, unprocessed lean animal protein sources such as eggs, and leafy green vegetables. Whilst these three food categories are nutrient dense, they have a high cost in Australia, leading to a lower nutrient density-to-cost ratio. Therefore, fresh fruit was penalized by the diet quality indices, where fruit was the only subcategory that did not improve in the modelled diet. The lack of improvement in fruit servings could also be due to it being selected the least often to replace discretionary foods in the modelling due to the hierarchy chosen (Supplementary Methods S2). The costs of low energy-dense, leafy green vegetables were inflated in this study, due to the need to match food categories per 100 kcal. There was also misalignment with the dietary guidelines for discretionary processed potatoes, which featured in the top quartile of nutrient dense, low-cost food categories, as they provide some dietary fiber and vitamin C [26]. To correct this misalignment, they were omitted from the modelled healthier, low-cost diet as per protocol, as this category is purely discretionary.

It is well established that the majority of ultra-processed foods are energy dense and nutrient poor, and that there is a link between a high consumption of ultra-processed foods and negative health outcomes [46,47,48,49]. This relationship may further be exacerbated by the manufacturing process, the presence of non-nutritive additives, and the disruption of the food matrix [50,51]. Despite a substantial reduction in ultra-processed foods in the healthier, low-cost diets, a quarter of the diet remained to consist of ultra-processed foods. The finding that a subset of low-cost, ultra-processed foods can be classified as nutrient dense, such as the ultra-processed wholegrain breads and ready-to-eat breakfast cereals with ≤20 g sugars/100 g, suggests misalignment between the NOVA scheme and nutrient density [19,20], especially when considering diet affordability. It has been proposed that lower SES groups readily select processed and ultra-processed foods for lower overall daily food costs, with a glass ceiling precluding their access to many unprocessed core foods [18]. The finding that nearly half of the foods in the modelled healthier, low-cost diet are processed and ultra-processed foods is consistent with previous research in the USA [16,19]. Consideration of the nutrient density and discretionary status of processed and ultra-processed foods is required to delineate the impact on diet quality and chronic disease, which may help to better inform public health messaging, consumer understanding of the classification, and clinical practice. As many populations and community groups rely on processing for food security [52], blanket dietary recommendations and public health strategies according to processing level alone could be reductionist and unrealistic. Specifically, the proportion of the category processed foods in the healthier, low-cost diets almost doubled. There is a need to revisit public health messaging about avoiding both processed and ultra-processed foods to ensure that we do not exclude low and middle SES families from accessing healthier more affordable core foods.

The current study has a number of strengths. The dietary data were nationally representative, and validated tools were used to assess nutrient density and diet quality. While previous Australian studies have used substitution modelling to examine nutritional interventions [53], or examine price differentials between current and healthy diets [12], this is the first study to use dietary substitution modelling to investigate the impact of nutrient dense, low-cost foods on parameters of both nutrition and cost. The assessment of food processing level in the modelled diets was also novel. Limitations of this study relate to the evidence that was used to inform the substitution modelling protocol. The generalizability of findings may be limited by the age of the dietary intake and nutrient composition data. Findings rely on the ranking of foods according to nutrient density, and thus the specific methods and tools used for its measurement. The NRF9.3 [36] was developed in the USA and selected as the preferred tool to measure nutrient density, rather than the Nutrient Profiling Scoring Criterion (NPSC) or the Health Star Rating (HSR) system, developed by Food Standards Australia New Zealand (FSANZ), and the Australasian governments, respectively. The NRF9.3 is a validated nutrient density tool, which has been used previously to compare the nutrient density of foods and assess the relationship nutrient density and cost [16,19,20,36]. However, it excludes some micronutrients, including all B-group vitamins, and it is important to consider that other nutrient density calculations may rank food categories differently as compared with the method used in this study. The lower cost of the healthier, low-cost diet may be partially due to it being lower in total energy; however, when matched for energy, the healthier, low-cost diet remained lower in cost than the current diet. The results are strengthened by similarities in the total number of servings per food category in both the current and healthier, low-cost diets, which confirms there were no differences in food volume. This study provides a theoretical proof-of-concept, and may not be translatable to individual dietary advice, particularly, due to the exclusion of mixed dishes in the modelling. It was not designed to model the composition of a nutritionally complete low-cost healthy diet, as this was outside the scope of this study. There is a need for research which embraces the complexity of the whole diet (e.g., including mixed dishes, take-away foods, and alcohol); and to examine whether individual foods in the top quartile of the nutrient density-to-cost ratio can be used to create a diet that meets national dietary recommendations.

## 5. Conclusions

This study provides evidence that nutrient dense, low-cost core foods can increase the quality and affordability of the Australian diet for both low and medium SES households. The nutrient dense, low-cost core foods were predominantly unprocessed and ultra-processed foods, which suggests that some non-discretionary ultra-processed foods may provide a beneficial source of nutrition when consumed within national dietary guideline recommendations. Future studies are needed to examine findings in other vulnerable groups, and to determine if foods can be modelled to meet national dietary recommendations.

## Figures and Tables

**Table 1:** Summary of the major steps involved in the protocol to model nutrient dense, low-cost diets.

Step	Purpose	Procedure
Step 1	Determine the nutrient density-to-cost ratio of food categories, and their NOVA processing levels and discretionary status	Calculate the nutrient density-to-cost ratio of the 57 food categories. Determine the NOVA processing level for each food category. Classify the nutrient density-to-cost ratio of the food categories into quartiles and map their NOVA processing level and discretionary status within the quartiles. Select the top quartile of non-discretionary nutrient dense, low-cost food categories for use in Step 2.
Step 2	Create nutrient dense, low-cost diets for low and medium SES households	Replace food categories in the bottom three quartiles of the current Australian diets of low and medium SES households with non-discretionary food categories in the top quartile of nutrient dense food categories (as identified in Step 1), using a substitution protocol. Determine the overall nutritional profile and total cost of current and modelled diets. Examine the diet quality and affordability of current and modelled diets.
Step 3	Determine the distribution of processed foods in the nutrient dense, low-cost diets	Determine the distribution of food categories by NOVA processing levels and discretionary status in the modelled diets (from Step 2).

**Table 2:** Demographics of low and medium SES reference households.

Indicators ^1^	Low SES Household	Medium SES Household	Low vs. Medium SES Household (*p*-Value) ^5^
	4-person reference household in the lowest quintile of SES position	4-person reference household in the middle quintile of SES position	
**Male (31–50 y)**			
*n*	281	324	
Age (years)	40.1 (5.8)	40.8 (5.9)	0.142
BMI ^2^			0.248
Underweight, *n* (%)	2 (0.8%)	2 (0.7%)	
Normal, *n* (%)	78 (32.0.%)	72 (25.2%)	
Overweight, *n* (%)	99 (40.6%)	139 (48.6%)	
Obese, *n* (%)	65 (26.6%)	73 (25.5%)	
**Female (31–50 y)**			
*n*	301	408	
Age (years)	40.2 (5.6)	40.1 (5.6)	0.814
BMI ^2^			0.009
Underweight, *n* (%)	5 (2.0%)	1 (0.3%)	
Normal, *n* (%)	89 (35.2%)	143 (42.6%)	
Overweight, *n* (%)	65 (25.7%)	101 (30.0%)	
Obese, *n* (%)	94 (37.1%)	91 (27.1%)	
**Male (9–13 y)**			
*n*	73	86	
Age (years)	10.9 (1.4)	10.9 (1.5)	1.000
BMI ^2^			0.219
Underweight, *n* (%)	3 (4.6%)	6 (8.7%)	
Normal, *n* (%)	37 (56.9%)	46 (66.7%)	
Overweight, *n* (%)	17 (26.2%)	14 (20.3%)	
Obese, *n* (%)	8 (12.3%)	3 (4.3%)	
**Female (4–8 y)**			
*n*	74	73	
Age (years)	5.9 (1.4)	5.7 (1.4)	0.610
BMI ^2^			0.270
Underweight, *n* (%)	5 (9.1%)	1 (1.7%)	
Normal, *n* (%)	40 (72.7%)	44 (75.9%)	
Overweight, *n* (%)	6 (10.9%)	10 (17.2%)	
Obese, *n* (%)	4 (7.3%)	3 (5.2%)	
**SES data**			
IRSAD quintile	1	3	
Equivalized disposable income (AUD) ^3^	AUD 399/week	AUD 902/week	
Adjusted household income (AUD) ^4^	AUD 798/week	AUD 1804/week	

IRSAD, Index of Relative Socioeconomic Advantage and Disadvantage; SES, socioeconomic status. ^1^ Data are mean (SD) unless otherwise indicated. ^2^ BMI data and categories were taken directly from the NNPAS microdata. ^3^ Sourced from the 2017–2018 Australian Survey of Income and Housing [38]. ^4^ Equivalized disposable income (2017–2018) multiplied by the OECD adjustment factor of 2 (for a household of 4) [11]. ^5^ For statistical significance, *p* < 0.005; *p*-values derived using two sample t-tests for continuous data and the chi-squared statistic for categorical data.

**Table 3:** Details for the top quartile of nutrient dense, low-cost food categories, including nutrient density/100 kcal, cost/100 kcal, the nutrient density to cost ratio, and NOVA processing classification.

Food Categories ^1^	NRF9.3/100 kcal ^2^	AUD/100 kcal	Nutrient Density-to-Cost Ratio ^3^	NOVA Processing Classification
**Vegetables**				
Orange/yellow vegetables	302.2 (126.0)	0.7 (0.1)	492.3 (304.5)	Unprocessed
Other vegetables	443.0 (160.6)	2.2 (1.4)	293.4 (171.9)	Unprocessed
Potatoes, unprocessed	97.1 (4.6)	0.5 (0.1)	217.0 (73.2)	Unprocessed
**Fruit**				
Fruit juice	273.7 (124.8)	0.5 (0.2)	650.1 (318.9)	Processed
Dried fruit	28.6 (9.0)	0.2 (0.1)	171.0 (80.1)	Unprocessed
**Cereals and grain foods**				
Rice, grains and flours	12.9 (4.6)	0.04 (0.0)	348.3 (196.5)	Unprocessed
Pasta and noodles, plain	20.7 (9.0)	0.1 (0.0)	304.6 (43.6)	Unprocessed
Hot porridge	22.6 (7.6)	0.2 (0.1)	290.9 (219.8)	Unprocessed
Breads, wholegrain and fortified	25.1 (3.1)	0.1 (0.0)	213.0 (57.7)	Ultra-processed
RTE breakfast cereals, fortified, sugars ≤20 g/100 g	25.3 (26.4)	0.1 (0.0)	200.1 (178.4)	Ultra-processed
RTE breakfast cereals, unfortified, sugars ≤20 g/100 g	24.9 (10.8)	0.2 (0.1)	176.1 (117.8)	Ultra-processed
**Dairy and dairy alternatives**				
Dairy milk alternatives	50.1 (30.7)	0.3 (0.1)	214.1 (161.3)	Ultra-processed
Dairy milk, reduced fat or skim	58.0 (13.0)	0.3 (0.1)	209.9 (79.0)	Unprocessed
**Meat and alternatives**				
Legumes and legume dishes	42.6 (5.6)	0.2 (0.0)	231.4 (50.0)	Processed
**Discretionary foods**				
Potatoes, processed	37.3 (4.9)	0.1 (0.0)	403.5 (109.3)	Ultra-processed

^1^ All data are mean (SD). ^2^ The NRF9.3 was calculated as described in references [16,36]. Food categories are defined as follows: ‘potatoes, unprocessed’, baked, boiled, grilled, or BBQ’d potatoes; ‘other vegetables’, all vegetables not otherwise categorized as potatoes, leafy green vegetables or orange/yellow vegetables; ‘breads, wholegrain and fortified’, commercial wholegrain breads fortified with iodine, Vitamin B1 and folic acid; ‘RTE breakfast cereals, fortified, sugars ≤20 g/100 g’, ready to eat breakfast cereals fortified with Vitamin B1, B2, B3, folic acid and iron, mean sugars content 10.5 g/100 g; ‘dairy milk alternatives’, commercial soy and rice-based milks; ‘potatoes, processed’, commercial oven-fries, wedges and hash-browns. ^3^ The nutrient density-to-cost ratio was calculated as the mean of the ratios for the representative foods chosen in each food category.

**Table 4:** The current Australian diet for each member of the reference household, for both low and medium SES positions.

Current Diet ^1^	Low SES Household					Medium SES Household				
	Male 31–50 y	Female 31–50 y	Male 9–13 y	Female 4–8 y	Total	Male 31–50 y	Female 31–50 y	Male 9–13 y	Female 4–8 y	Total
*n*	281	301	73	74		324	408	86	73	
**Diet cost and affordability**										
Cost of diet per week (AUD)	36.8 (1.2)	28.9 (1.0)	35.3 (1.9)	28.7 (1.5)	129.7 (2.9)	39.2 (1.2)	32.7 (0.9)	38.2 (2.0)	30.8 (1.5)	140.9 (2.9)
Diet affordability (%) ^2^	-	-	-	-	16.3	-	-	-	-	7.8
**Total energy (kcal/day)**	1715.8 (61.7)	1238.7 (38.8)	1697.1 (89.7)	1495.0 (74.4)	6146.6 (137.5)	1680.3 (45.7)	1378.7 (35.1)	1868.8 (80.9)	1430.8 (74.2)	6358.6 (124.0)
**Food groups (servings/day ^3^)**										
Vegetables	1.8 (0.2)	1.6 (0.1)	1.1 (0.2)	0.6 (0.1)	5.2 (0.3)	1.9 (0.1)	1.7 (0.1)	0.8 (0.2)	1.0 (0.2)	5.4 (0.3)
Fruit	1.2 (0.1)	1.2 (0.1)	1.6 (0.2)	1.4 (0.2)	5.5 (0.3)	1.3 (0.1)	1.2 (0.1)	1.7 (0.2)	2.0 (0.2)	6.2 (0.3)
Grain (cereal) foods	3.3 (0.2)	2.4 (0.1)	3.0 (0.3)	2.8 (0.2)	11.5 (0.4)	3.4 (0.2)	2.5 (0.1)	3.6 (0.2)	2.7 (0.2)	12.2 (0.4)
Lean meats and alternatives	1.3 (0.1)	0.9 (0.1)	0.7 (0.1)	0.5 (0.1)	3.4 (0.2)	1.4 (0.1)	1.1 (0.1)	0.7 (0.1)	0.3 (0.1)	3.5 (0.2)
Dairy and dairy alternatives	1.4 (0.1)	1.1 (0.1)	1.6 (0.2)	1.6 (0.1)	5.7 (0.3)	1.3 (0.1)	1.1 (0.1)	1.7 (0.2)	1.5 (0.2)	5.7 (0.3)
Discretionary foods	5.7 (0.3)	4.0 (0.2)	5.9 (0.5)	4.3 (0.4)	19.9 (0.7)	5.8 (0.3)	4.3 (0.2)	7.0 (0.5)	4.6 (0.3)	21.6 (0.7)
**Macronutrients (% of energy)**										
Protein	12.9 (0.3)	13.0 (0.3)	12.0 (0.5)	11.4 (0.4)	12.7 (0.2)	13.7 (0.3)	13.2 (0.3)	11.3 (0.3)	11.0 (0.3)	13.0 (0.2)
Total fat	40.8 (0.9)	41.7 (0.8)	39.6 (1.4)	41.5 (1.4)	41.1 (0.5)	39.6 (0.7)	41.8 (0.7)	40.2 (1.3)	43.5 (1.3)	41.0 (0.5)
Saturated fat	15.7 (0.5)	16.4 (0.5)	15.7 (0.8)	16.2 (0.7)	16.1 (0.3)	15.3 (0.4)	16.5 (0.4)	15.9 (0.7)	17.9 (0.8)	16.1 (0.3)
Monounsaturated fat	16.3 (0.4)	16.3 (0.4)	15.7 (0.7)	16.4 (0.7)	16.2 (0.3)	15.8 (0.3)	16.2 (0.3)	15.8 (0.6)	16.6 (0.5)	16.0 (0.2)
Polyunsaturated fat	5.8 (0.2)	6.1 (0.2)	5.3 (0.3)	6.0 (0.4)	5.9 (0.1)	5.7 (0.2)	6.1 (0.2)	5.5 (0.3)	5.8 (0.3)	5.9 (0.1)
Total Carbohydrates	44.7 (0.9)	43.8 (0.8)	46.5 (1.4)	45.3 (1.4)	44.6 (0.5)	45.0 (0.7)	43.3 (0.6)	46.8 (1.3)	43.9 (1.2)	44.3 (0.5)
Total sugars	22.1 (0.9)	22.4 (0.7)	16.8 (1.0)	17.0 (0.9)	21.2 (0.4)	22.2 (0.7)	20.4 (0.5)	17.8 (0.8)	17.7 (0.9)	20.6 (0.4)
Added sugars	10.8 (0.6)	10.4 (0.4)	7.8 (0.6)	8.1 (0.5)	10.1 (0.3)	10.6 (0.3)	9.3 (0.3)	9.2 (0.5)	8.2 (0.5)	9.7 (0.2)
Free sugars	15.2 (0.8)	15.0 (0.6)	10.2 (0.9)	10.5 (0.8)	14.1 (0.4)	15.2 (0.5)	12.8 (0.5)	11.5 (0.7)	10.5 (0.7)	13.4 (0.3)
Starch	22.6 (0.7)	21.4 (0.6)	29.4 (1.1)	28.2 (1.0)	23.3 (0.4)	22.7 (0.5)	22.8 (0.5)	28.8 (1.1)	26.0 (0.8)	23.6 (0.4)
Dietary fiber (g/day)	18.2 (0.8)	13.9 (0.6)	16.9 (1.0)	15.4 (0.8)	64.5 (1.6)	18.5 (0.6)	15.6 (0.5)	19.7 (1.1)	16.3 (0.9)	70.1 (1.6)

^1^ All data are mean (SEM) per day, of all diets used for analysis, unless otherwise stated. ^2^ Diet affordability is the mean diet cost per week expressed as a percentage of equivalized disposable income (as shown in Table 3). ^3^ Servings per day were calculated on the basis of the servings sizes provided in the Australian Guide to Healthy Eating [39]. SES, socioconomic status.

**Table 5:** The theoretical modelled healthier, low-cost diet for each member of the reference household, for both low and medium SES positions.

Healthier, Low-Cost Modelled Diet ^1^	Low SES Household					Medium SES Household					Current vs. Healthier Diets *p*-Value ^3^	
	Male 31–50 y	Female 31–50 y	Male 9–13 y	Female 4–8 y	Total	Male 31–50 y	Female 31–50 y	Male 9–13 y	Female 4–8 y	Total	Low SES	Medium SES
*n*	281	301	73	74		324	408	86	73			
**Diet cost and affordability**												
Cost of diet per week, (AUD)	27.8 (1.0)	21.6 (0.7)	27.2 (1.6)	21.0 (1.0)	97.6 (2.2)	28.4 (0.9)	22.8 (0.6)	28.8 (1.5)	22.7 (1.2)	102.6 (2.2)	<0.001	<0.001
Diet affordability (%) ^2^					12.2					5.7		
**Total energy (kcal/day)**	1569.2 (64.7)	1108.1 (42.8)	1415.3 (89.3)	1210.3 (66.5)	5302.9 (135.7)	1519.8 (49.8)	1222.6 (37.7)	1543.7 (77.2)	1212.0 (80.0)	5498.1 (127.5)	<0.001	<0.001
**Food groups (servings/day ^4^)**												
Vegetables	5.7 (0.2)	4.4 (0.2)	5.4 (0.4)	3.9 (0.2)	19.4 (0.5)	5.7 (0.2)	4.6 (0.1)	6.0 (0.4)	4.2 (0.3)	20.5 (0.5)	<0.001	<0.001
Fruit	1.2 (0.1)	1.2 (0.1)	1.6 (0.2)	1.5 (0.2)	5.6 (0.3)	1.4 (0.1)	1.3 (0.1)	1.8 (0.2)	2.0 (0.2)	6.5 (0.3)	0.068	0.001
Grain and cereal foods	3.5 (0.2)	2.5 (0.1)	3.2 (0.3)	2.9 (0.2)	12.1 (0.4)	3.7 (0.2)	2.6 (0.1)	3.8 (0.2)	2.8 (0.2)	12.8 (0.4)	<0.001	<0.001
Lean meats and alternatives	2.3 (0.2)	1.5 (0.1)	1.6 (0.2)	1.2 (0.2)	6.5 (0.4)	2.3 (0.1)	1.8 (0.1)	1.5 (0.2)	1.3 (0.2)	7.0 (0.3)	<0.001	<0.001
Dairy and dairy alternatives	1.9 (0.1)	1.4 (0.1)	2.0 (0.2)	1.7 (0.2)	7.0 (0.3)	1.8 (0.1)	1.6 (0.1)	2.1 (0.2)	1.7 (0.2)	7.2 (0.3)	<0.001	<0.001
Discretionary foods	0.0 (0.0)	0.0 (0.0)	0.0 (0.0)	0.0 (0.0)	0.0 (0.0)	0.0 (0.0)	0.0 (0.0)	0.0 (0.0)	0.0 (0.0)	0.0 (0.0)	<0.001	<0.001
**Macronutrients (% of energy)**												
Protein	12.7 (0.4)	12.9 (0.4)	12.4 (0.7)	10.4 (0.5)	12.5 (0.3)	12.9 (0.3)	12.1 (0.3)	11.1 (0.5)	9.7 (0.5)	12.1 (0.2)	<0.001	<0.001
Total fat	29.8 (1.6)	31.0 (1.5)	27.8 (3.7)	33.5 (2.7)	30.5 (1.1)	26.9 (1.4)	29.8 (1.2)	32.8 (2.7)	37.5 (2.7)	29.7 (1.1)	<0.001	<0.001
Saturated fat	3.8 (0.2)	4.0 (0.2)	3.7 (0.3)	3.9 (0.3)	3.9 (0.1)	3.5 (0.2)	3.9 (0.1)	4.1 (0.3)	4.6 (0.3)	3.8 (0.1)	<0.001	<0.001
Monounsaturated fat	11.6 (0.7)	12.1 (0.7)	10.8 (1.2)	13.1 (1.2)	11.9 (0.5)	10.4 (0.6)	11.6 (0.5)	12.9 (1.1)	14.9 (1.2)	11.6 (0.5)	<0.001	<0.001
Polyunsaturated fat	12.1 (0.7)	12.6 (0.7)	11.2 (1.2)	14.2 (1.2)	12.4 (0.5)	10.9 (0.6)	12.0 (0.5)	13.5 (1.2)	15.5 (1.2)	12.0 (0.5)	0.093	0.232
Total Carbohydrates	52.2 (1.2)	51.1 (1.2)	55.0 (2.1)	51.8 (2.1)	52.0 (0.9)	54.6 (1.1)	53.1 (1.0)	51.6 (2.1)	48.8 (2.1)	53.1 (0.8)	0.209	0.118
Total sugars	21.1 (1.0)	23.2 (0.9)	24.3 (1.6)	22.0 (1.5)	22.4 (0.6)	22.4 (0.9)	23.9 (0.8)	20.2 (1.2)	23.1 (1.4)	23.0 (0.6)	<0.001	<0.001
Added sugars	1.3 (0.1)	1.2 (0.1)	1.7 (0.2)	1.5 (0.2)	1.3 (0.1)	1.5 (0.1)	1.4 (0.1)	1.8 (0.2)	1.4 (0.2)	1.5 (0.1)	<0.001	<0.001
Free sugars	1.6 (0.1)	1.6 (0.1)	2.6 (0.4)	2.1 (0.2)	1.8 (0.1)	2.3 (0.3)	1.8 (0.1)	2.5 (0.2)	1.9 (0.2)	2.1 (0.1)	<0.001	<0.001
Starch	30.8 (1.0)	27.6 (0.9)	30.5 (1.6)	29.6 (1.5)	29.3 (0.6)	31.9 (0.8)	28.8 (0.7)	31.1 (1.7)	25.5 (1.3)	29.9 (0.6)	<0.001	<0.001
Dietary fiber (g/day)	35.2 (2.3)	39.7 (1.5)	28.2 (1.7)	28.6 (1.0)	131.8 (3.4)	37.7 (1.9)	41.3 (1.3)	29.1 (1.6)	139.9 (1.2)	139.9 (1.9)	<0.001	<0.001

^1^ All data are mean (SEM) per day, of all diets used for analysis, unless otherwise stated. ^2^ Diet affordability is the mean diet cost per week expressed as a percentage of equivalized disposable income (as shown in Table 3). ^3^ *p*-values comparing diets from a general linear model for change in values with SES, age-sex group, and their interaction as fixed factors. For statistical significance, *p* < 0.005. ^4^ Servings per day were calculated based on the recommendations provided in the Australian Guide to Healthy Eating [39]. SES, socioeconomic status.

**Table 6:** Distribution of NOVA processing levels and food category types throughout the current and healthier diets for both low and medium SES households.

Category Distribution ^1^	Low SES Diets		Medium SES Diets		Current vs. Healthier Diets *p*-Value ^2^	
	Current	Healthier	Current	Healthier	Low SES	Medium SES
*n*	729	729	891	891		
**Unprocessed**	31.3% (0.8)	59.4% (0.7)	32.2% (0.7)	58.0% (0.6)	<0.001	<0.001
Vegetables	20.6% (1.1)	66.3% (0.9)	21.2% (0.9)	67.1% (0.8)	<0.001	<0.001
Fruit	21.3% (1.1)	13.7% (0.7)	23.5% (1.0)	14.4% (0.6)	<0.001	<0.001
Pasta, rice and other grains	9.6% (0.8)	7.1% (0.6)	8.7% (0.7)	5.9% (0.5)	0.010	0.001
Hot porridge	1.2% (0.3)	0.9% (0.2)	2.3% (0.3)	1.5% (0.2)	0.405	0.042
Red meat, poultry and eggs	14.8% (0.9)	0.0% (0.0)	17.8% (0.9)	0.0% (0.0)	<0.001	<0.001
Fish	1.1% (0.3)	0.0% (0.0)	2.1% (0.3)	0.0% (0.0)	<0.001	<0.001
Nuts and seeds	3.7% (0.5)	0.0% (0.0)	3.5% (0.4)	0.0% (0.0)	<0.001	<0.001
Full-fat milk	18.9% (1.2)	0.0% (0.0)	12.4% (0.8)	0.0% (0.0)	<0.001	<0.001
Reduced fat milk	7.3% (0.7)	12.1% (0.6)	7.2% (0.6)	11.1% (0.5)	<0.001	<0.001
Yoghurt, full fat	1.5% (0.3)	0.0% (0.0)	1.3% (0.2)	0.0% (0.0)	<0.001	<0.001
**Processed culinary**	1.6% (0.2)	3.5% (0.3)	1.6% (0.2)	3.1% (0.2)	<0.001	<0.001
Oils	13.3% (3.0)	100.0% (0.0)	12.5% (2.4)	100.0% (0.0)	<0.001	<0.001
Discretionary fats	86.7% (3.0)	0.0% (0.0)	87.5% (2.4)	0.0% (0.0)	<0.001	<0.001
**Processed**	6.9% (0.4)	12.7% (0.5)	6.6% (0.3)	14.3% (0.4)	<0.001	<0.001
Fruit	27.5% (2.2)	11.5% (1.2)	27.6% (1.9)	9.7% (0.9)	<0.001	<0.001
Tinned fish	5.7% (1.1)	0.0% (0.0)	7.5% (1.1)	0.0% (0.0)	<0.001	<0.001
Cheese	25.1% (2.0)	0.0% (0.0)	28.9% (1.9)	0.0% (0.0)	<0.001	<0.001
Legumes and legume dishes	4.6% (1.0)	88.5% (1.2)	4.6% (0.9)	90.3% (0.9)	<0.001	<0.001
Discretionary	37.1% (2.3)	0.0% (0.0)	31.4% (1.9)	0.0% (0.0)	<0.001	<0.001
**Ultra-processed**	60.2% (0.9)	24.4% (0.7)	59.6% (0.7)	24.7% (0.6)	<0.001	<0.001
Vegetable dishes	5.3% (0.6)	0.0% (0.0)	6.4% (0.5)	0.0% (0.0)	<0.001	<0.001
Commercial fruit, in syrup	0.1% (0.1)	2.3% (0.4)	0.1% (0.0)	2.5% (0.4)	<0.001	<0.001
Pasta and noodles, with additions	0.1% (0.0)	0.0% (0.0)	0.0% (0.0)	0.0% (0.0)	0.1681	0.158
Wholegrain breads	7.6% (0.7)	57.3% (1.5)	7.5% (0.5)	52.7% (1.3)	<0.001	<0.001
Refined breads and flours	16.6% (0.8)	0.0% (0.0)	14.4% (0.7)	0.0% (0.0)	<0.001	<0.001
RTE breakfast cereals, ≤20 g sugars/100 g	6.0% (0.5)	15.1% (1.1)	7.8% (0.5)	18.2% (1.0)	<0.001	<0.001
Meat alternatives	0.3% (0.2)	0.0% (0.0)	0.2% (0.1)	0.0% (0.0)	0.0891	0.129
Milk, and milk-based beverages	2.9% (0.4)	0.0% (0.0)	2.7% (0.3)	0.0% (0.0)	<0.001	<0.001
Dairy milk alternatives	0.4% (0.2)	23.0% (1.2)	0.6% (0.2)	24.3% (1.1)	<0.001	<0.001
Yoghurt, reduced fat	0.8% (0.2)	0.0% (0.0)	1.1% (0.2)	0.0% (0.0)	<0.001	<0.001
Cheese	1.9% (0.2)	0.0% (0.0)	1.3% (0.1)	0.0% (0.0)	<0.001	<0.001
Margarines and margarine-like spreads	3.9% (0.3)	2.3% (0.4)	3.3% (0.2)	2.3% (0.3)	<0.001	0.007
Discretionary	54.1% (1.1)	0.0% (0.0)	54.6% (0.9)	0.0% (0.0)	<0.001	<0.001

^1^ All data are mean (SEM) percentage of all diets used for analysis. ^2^ For statistical significance, *p* < 0.005. Comparisons were calculated via unpaired Student’s *t*-test. Food categories are defined as follows: ‘discretionary fats’ includes butter and cream; ‘processed fruit’ includes commercial fruit in juice and fruit juice; ‘ultra-processed vegetable dishes’, include commercially produced coleslaws, potato salad, and ‘heat and eat’ vegetable dishes; ‘wholegrain breads and flours’ include commercial wholegrain breads both unfortified and fortified with iodine, Vitamin B1, and folic acid; ‘refined breads and flours’ include commercial white breads both unfortified and fortified with iodine, Vitamin B1, and folic acid ‘ready-to-eat (RTE) breakfast cereals, sugars ≤20 g/100 g’ include ready-to-eat breakfast cereals both unfortified and fortified with Vitamin B1, B2, B3, folic acid, and iron, mean sugars content 11.9 g/100 g; ‘meat alternatives’ include commercially produced vegetarian sausages and vegetarian burgers; ‘milk and milk-based beverages include fortified milk drinks and flavored milk; ‘dairy milk alternatives’ include commercially-produced rice and soy milk.

## Data Availability

Access to the AUSNUT 2011–13 data file can be found at https://www.foodstandards.gov.au/science/monitoringnutrients/ausnut/pages/default.aspx (accessed on 22 October 2020).

## Supplementary Materials

The following are available online at https://www.mdpi.com/article/10.3390/ijerph18115771/s1. Table S1: Details and descriptions of all food categories, including all 8-digit codes included in each food category, Methods S1: The full calculation of NRF9.3, adapted for the Australian Nutrient Reference Values, Table S2: Reference values from the NRF9.3 and corresponding Australian Nutrient Reference Values used in the current study, Methods S2: Rules used to develop the substitution modelling protocol, Table S3: Nutrient density and NOVA processing level of all 57 food categories, Table S4: Micronutrient analysis of the current and healthier, low-cost diets for each member of both low and medium socioeconomic households, Table S5: Individual component scores for the HEIFA-2013 and DGI-CA, for each member of both low and medium SES households.
