# Supplementary material for: Diet and Economic Modelling to Improve the Quality and Affordability of the Australian Diet for Low and Medium Socioeconomic Households
Source: Int J Environ Res Public Health. 2021 May 27;18(11):5771. doi: 10.3390/ijerph18115771 (PMC8198747; doi:10.3390/ijerph18115771)
Supplement: Supplementary file 1 [file ijerph-18-05771-s001.zip › ijerph-1193431-supplementary.pdf]

Table S1. Details and descriptions of all food categories, including all 8-digit codes included in each food category [1].

Major food group (2-digit code)	Food category	Description	8-digit codes included
Nonalcoholic beverages (11)	Fruit and vegetable juice	Commercial and non-commercial juices	11301001-11306003
	Sweetened beverages	Fruit drinks, cordials, soft drinks, flavoured mineral waters, sports drinks, and energy drinks	11307001-11604003
	Liquid breakfast, fortified beverages (like Milo)	Milo prepared with milk, unfortified beverage flavourings prepared with milk, beverage bases unmixed, and breakfast cereal beverages	11801001-11806002
Cereals and cereal products (12)	Rice, grains and flours	Oats, rice, quinoa and couscous without additions	12101001-12104002
	Breads and rolls, wholegrain, fortified [2,3]	Bagels, bread, English muffins, and flat breads with mandatory or additional fortification, all grains including gluten-free varieties	12204001-12204018; 12207001-12207022; 12210001-12210010; 12205; 12208001-12208008; 12211
	Breads and rolls, refined grain, fortified	White (including gluten-free) bagels, bread, English muffins, and flat breads with mandatory or additional fortification	12201001-12201029; 12202001-12202010
	Breads and rolls, wholegrain (all grains), unfortified	Bagels, bread, English muffins, and flat breads with no fortification, all grains including gluten-free varieties	12301004-12301005; 12302004-12302005; 12302008-12302011; 12303001-12303007; 12206001-12206006; 12209001-12209009; 12212001-12212004; 12213001-12213006
	Breads and rolls, refined grain (white), unfortified	White (including gluten-free) bagels, bread, English muffins, and flat breads with no fortification	12301001-12301003; 12302001-12302003; 12302006-12302007; 12302012-12302014; 12203001-12203023; 12304001-12304008; 12307001-12307004; 12305001-12305006
Cereals and cereal products (12); Cereal-based products and dishes (13); Fruit products and dishes (16); Milk products and dishes (19); Sugar products and dishes (27)	Desserts	Sweet buns, sweet biscuits, cakes, cupcakes, banana breads, cake-style muffins with chocolate, pudding, pie, danishes, éclairs, doughnuts, ice cream, toppings, pavlova, and ice confections	12305007-12305010; 12306001-12306009; 13101001-13107001; 13301001-13303077; 13304011-13304014; 13304018-13304019; 13305001-13306029; 13308003; 13309001-

			13309004; 13402001-13403021; 13605001-13605011; 16901001-16901007; 19501001-19508011; 19601001-19602007; 19701001-19702007; 27301001-27304015; 27103001-27103006
	Pasta and noodles, plain	Pasta and noodles without additions.	12401001-12403007
	Pasta and noodles, with additions	Filled pasta (meat, spinach, cheese, vegetables)	12404001-12404004
Cereals and cereal products (12)	Breakfast cereal, RTE, fortified, sugars ≤20g/100g [4]	Fortified ready-to-eat breakfast cereals and muesli varieties, with or without fruit and nuts, and lower in sugar. *E.g.,* Weet-bix, Just Right	12506001-12516015; 12512001-12512006; 12502001-12502007; 12502011-12502012; 12504001-12504008; 12509001-12509007; 12502008-12502010; 12515001-12515027
	Breakfast cereal, RTE, fortified, sugars >20g/100g	Fortified ready-to-eat breakfast cereals and muesli, with or without fruit and nuts high in sugar. E.g., cocoa puffs, Sultana Bran	12507001-12507002; 12513001-12513010; 12510001-12510003
	Breakfast cereal, RTE, unfortified, sugars ≤20g/100g	Unfortified ready-to-eat breakfast cereals and muesli varieties, lower in sugar. E.g., Puffed wheat, natural muesli	12501001-12501003; 12501007; 12503001-12503002; 12505001-12505006; 12511005; 12514010-12516001-12516003; 12514001; 12514004; 12514007; 12514018-12414024; 12516003
	Breakfast cereal, RTE, unfortified, sugars >20g/100g	Unfortified ready-to-eat breakfast cereals and muesli high in sugar. E.g., muesli with sugar and syrup added	12503003; 12505007; 12511002-12511004; 12514002; 12414003; 12514005; 12514017; 12515006; 12516001-12516002
	Hot porridge	Porridge with and without (milk, sugar, honey and dried fruit)	12601001-12601018; 12602001-12602003
Cereal-based products and dishes (13)	Savoury biscuits	Crackers, wafers, rice cakes, and crispbreads,	13201001-13205003
	Muffins and scones	Un-iced cake-style muffins and scones	13304001-13304010; 13304015-13304017; 13304020-13304021; 13307001-13308002
	Batter-based products	Crepes, pancakes, pikelet, waffles, fritters, Yorkshire pudding, crumpets without additions.	13601001-13604010; 13606001-13606003

Fats and oils (14)	Solid fats	Butter and other solid fats	14101001-14102002; 14501001-14503001; 14602001
	Oils	Cooking oils	14401001-14403001
	Margarines and margarine-like spreads	Dairy blends, margarines and table spreads	14201001-14203001; 14301001-14307011; 14601001-14601002
Fish and seafood products and dishes (15)	Fish, unprocessed, without additions [5]	Fish (baked, boiled, roasted, smoked). Crabs, prawns, scallops, squid	15101001-15503005
	Fish, processed, battered or crumbed	Fish and shellfish, battered and fried	15501001-15504016
	Fish, processed, tinned	Canned and processed fish	15401001-15402003
Fruit products and dishes (16)	Fruit	Raw fruits and fruit salads	16101001-16101020; 16103001-16103011; 16105001-16105002; 16201001-16201012; 16301001-16303005; 16401001-16401011; 16403001-16403006; 16501001-16504012; 16601001-16701032
	Commercial fruit, in brine or juice	Canned fruits (drained, with juice)	16102001; 16104001-16104003; 16304001; 16402001-16402003; 16404001-16404003; 16505001-16505003; 16505005-16505007; 16702001; 16702014-16702105
	Commercial fruit, in syrup	Canned fruits (in syrup)	16104004-16104013; 16202001-16202010; 16304002-16304004; 16402004-16402013; 16404004-16404018; 16505004; 16505008-16505010; 16702013
	Dried fruit	Prunes, raisins, sultanas, dates, figs	16801001-16802014
Egg products and dishes (17)	Eggs, without additions	Eggs, plain (poached, hard-boiled)	17101001-17103006
Meat, poultry and game products and dishes (18)	Red meat, fat fully-trimmed	Beef, lamb, pork, veal, kangaroo with fat fully removed	18101005; 18101012-18101016; 18101025; 18101032-18101035; 18101040-18101044; 18101047-18101057; 18101064-18101071; 18101087-18101088; 18101092; 18101103-18101109; 18101121-18101128; 18101148-18101154; 18101165-18101171; 18101180-18101186; 18101201-

		18101208; 18101217-18101219; 18101233-18101235; 18101244-18101247; 18101253-18101259; 18102004; 18102007-18102012; 18102015-18102023; 18102042-18102043; 18102052-18102055; 18102060; 18102064-18102065; 18102072-18102073; 18102081-18102088; 18102098-18102101; 18102114-18102115; 18102122-18102123; 18102137-18102138; 18102145-18102146; 18102054-18102157; 18102168-18102170; 18102180-18102182; 18102194-18102195; 18102202-18102203; 18103006-18103010; 18103015-18103035; 18103040-18103041; 18103057-18103064; 18103081-18103083; 18103090-18103092; 18103106-18103107; 18103110-18103113; 18103118-18103119; 18103123-18103124; 18103126-18103130; 18103141-18103144; 18104005-18104007; 18104014-18104017; 18104022-18104028; 18104035-18104036; 18104043-18104044; 18201001-18201008; 18202013-18202021
Red meat, untrimmed or semi-trimmed	Beef, lamb, pork, veal, kangaroo with all fat remaining or partially removed	18101007; 18101017-18101023; 18101027; 18101036-18101037; 18101045-18101046; 18101058-18101061; 18101072-18101086; 18101089-18101090; 18101094-18101096; 18101110-18101114; 18101129-18101147; 18101155-18101164;

		18101172-18101177; 18101187-18101196; 18101209-18101210; 18101220-18101229; 18101236-18101240; 18101249-18101251; 18101261-18101273; 18102006; 18102013; 18102025-18102039; 18102044-18102049; 18102056-18102057; 18102061; 18102066-18102069; 18102074-18102077; 18102089-18102095; 18102102-18102109; 18102116-18102119; 18102124-18102134; 18102139-18102143; 18102148-18102151; 18102158-18102165; 18102171-18102179; 18102183-18102185; 18102196-18102197; 18102204-18102207; 18103001-18103004; 18103011-18103013; 18103036-18103039; 18103042-18103048; 18103066-18103078; 18103084-18103087; 18103093-18103103; 18103108; 18103114-18103115; 18103120-18103122; 18103125; 18103131-18103140; 18103145; 18104008-18104011; 18104018-18104019; 18104029-18104030; 18104038-18104040; 18104045-18104046; 18202001-18202012
Poultry	Chicken, duck, feathered game	18301001-18303010
Ultra-processed meats	Sausages (beef, pork, chicken), frankfurters, saveloys)	18501001-18503009
Processed meat	Bacon, ham, prosciutto, salami, corned beef, turkey deli-sliced, spam, and beef jerky	18601001-18607001

Milk products and dishes (19)	Dairy milk, full fat	Full fat fluid milk, condensed milk, and milk powder	19101001-19101008; 19102001; 19106001; 19106004; 19107001; 19108001-19108003; 19109001-19109003
	Dairy milk, reduced fat or skim	Reduced fat fluid milk, condensed milk, and milk powder	19103001-19105005; 19106002-19106003; 19106005; 19107012
	Yoghurt, full fat	Full fat and high fat yoghurt, plain and sweetened	19201001-19201005; 19204001-19206003; 19211001-19211002; 19211011; 19211013; 19212001-19212012
	Yoghurt, reduced fat	Reduced fat yoghurt, plain and sweetened	19202001-19202003; 19203001; 19207001-19209010; 19210001-19210003; 19211003-19211010; 19211012; 19211014-19211015
	Cream	Cream, sour cream, and whipped cream	19301001-19306004
	Cheese	Blocks and grated, all varieties	19401001-19405003
	Ultra-processed cheese	Cheese slices ('plastic' cheese)	19406001-19408003
	Flavoured milk	Iced coffee, milk with flavouring, milkshakes, thick shakes, and fruit smoothies with milk	19801001-19806007
Dairy & meat substitutes (20)	Dairy milk substitutes	Soy beverages and rice beverages including smoothies and milkshakes, and soy based yogurts	20101001-20502003
	Meat substitutes and dishes	Vegetarian sausages, tofu, tempeh, veggie burgers, stir-fry with tofu	20601001-20701004
Seed and nut products and dishes (22)	Seeds and nuts	Chia seeds, nuts (peanuts, almonds, pistachio, pine), peanut butter, satay sauce, and coconut milk	22101001-22205019
Savoury sauces and condiments (23)	Dips	Dairy-based dips, hummus and guacamole	23501001-23501010; 23502001-23504003
Vegetable products and dishes (24)	Potatoes, unprocessed	Baked, boiled, fried, grilled, or BBQed potatoes.	24101001-24101065
	Potatoes, processed	Fries, hash browns, and wedges.	24102001-24102036
	Orange/yellow vegetables	Carrots, orange sweet potato, pumpkin	24301001-24301013; 24302034-24302042; 24701001-24701028
	Green leafy vegetables	Lettuce, rocket, spinach	24401001-24401025
	Other vegetables	Broccoli, cabbage and cauliflower, leeks, onions, peas	24201001-24202017; 24302001-24302033;

		and beans, beetroot, root vegetables that are not orange or yellow, zucchini, tomato, mushrooms, onions and leeks	24302049-24302054; 24402001-24602009; 24702001-24802026
	Vegetable dishes	Vegetable casseroles, vegetable curries with (rice, noodles), vegetable stir-fry, vegetable salads with additions (cheese, fruit, nuts meat, eggs), coleslaw, and potato salad	24901001-24905023
Legume and pulse products and dishes (25)	Legumes and legume dishes	Beans, lentils, baked beans plain and in (sauces, curry, salads and casseroles)	25101001-25202016
Snack foods (26)	Savoury snacks	Potato crisps, potato straws, corn chips, popcorn with additions (salt, butter), extruded snacks, snack mixes, and pretzels	26101001-26401016
Sugar products and dishes (27)	Sweet spreads and syrups	Fruit jams, chocolate/hazelnut spread; Sugar, honey, and maple syrups	27101001-27102005; 27201001-27205003
Confectionery and cereal, nut, fruit, seed bars (28)	Sweet snacks	Muesli and cereal bars (fruit bars, fruit leathers, muesli bars with and without coatings or chocolate chips), chocolate, bars, licorice, honeycomb, lollies, marshmallows	28101001-28104001; 28201001-28202008; 28301001-28305004; 28401001-28405003

[1] Each food category can be matched to a major food group, at the 2-digit code level. The 2-digit codes pertaining to each major food group are given in brackets. Some of the food categories align with more than one major food group (2-digit code); details are provided where necessary. The aggregation of the sub-minor food groups into food categories was carried out based on the nutritional and processing properties of each sub-major food group (3-digit code), minor food group (5-digit code), and individual food (8-digit code). [2] Where specified, fortified foods were grouped together; non-fortified foods were thus also grouped together. [3] Where specified, wholegrain versions of a food were grouped together; thus, refined versions of a food were also grouped together. [4] Where applicable, the AUSNUT classifications based on nutritional properties were used: High or moderate fat foods were grouped together, and reduced or skimmed versions of a food were grouped together; Foods with a similar sugar content were grouped together; Foods high in sugar and classified as discretionary were grouped together. [5] Where possible, foods within each level of processing (NOVA classification) were grouped together. A selection of 2-digit codes were excluded. These were: mixed dishes and takeaway foods, due to them containing more than one food category and therefore creating added complexity that would be unfeasible to model; breads and rolls with flour not defined, as it was not possible to determine the status as wholegrain or refined; organ meats and offal, due to a low frequency of consumption; tea and coffee, due to contributing negligible calories to the diet.; water, due to contributing negligible calories to the diet; alcohol, due to contributing negligible nutrients to the diet; supplements, due to contributing negligible calories to the diet; infant formula and baby foods, due to no household member being an infant or baby.

Supplementary Methods S1. The calculation of NRF9.3, adapted to the Australian Dietary Guidelines and Nutrient Reference Values.

The NRF9.3/100 kcal provides an algorithm to assess the proportion of nutrient requirements that are provided by a food, in relation to the energy provided by the food. There are nine positively weighted nutrients (protein, dietary fibre, calcium, iron, potassium, magnesium, and vitamins A, C and E), and three negatively weighted nutrients (saturated fat, added sugar, and sodium).

The NRF9.3/100 kcal was calculated for each of the three representative foods in each of the 57 food categories (171 foods in total), with daily values calculated as a percentage per 100 kcal and capped at 100%.

The complete calculation for the NRF9.3/100 kcal is:

1. NR9 subscore:

$$NR9/serving = \sum_{1\text{–}9} (nutrient/daily\ value) \times 100$$

$$NR9/100\ kcal = \sum_{1\text{–}9} (nutrient/daily\ value)/(kcal\ per\ serve) \times 100$$

2. LIM subscore:

$$LIM/serving = \sum_{1\text{–}3} (nutrient/maximum\ recommended\ value) \times 100$$

$$LIM/100\ kcal = \sum_{1\text{–}3} (nutrient/maximum\ recommended\ value)/(kcal\ per\ serve) \times 100$$

3. Composite NRF9.3/100 kcal

$$NRF9.3/100\ kcal = NR9_{100\ kcal} - LIM_{100\ kcal}$$

Since the nutrients used in the NRF9.3 are mostly in line with the Nutrient Profiling Scoring Criterion (NPSC, Food Standards Australia and New Zealand), it was deemed unnecessary to change all the nutrients included in the Australian-modified NRF 9.3 [37]. However, the full set of reference values were determined based on the following additional criteria:

a. The reference used was an adult aged 18–50 years.

b. When men and women had a different RDI or AI, the higher value was chosen. For example, the recommended dietary intake (RDI) for iron for men was 8 mg/day, and for women was 18 mg/day; therefore, the higher value of 18 mg/day was chosen.

c. When men and women had a different maximum recommended value (MRV), the lower value was chosen. For example, the saturated fat MRV is 9% of dietary energy. The total dietary energy was based on the lowest average daily energy requirement for a sedentary working female (physical activity level [PAL] of 1.6) [37].

d. The upper limit for Added Sugar was based on the WHO recommendations that added sugar should not be more than 10% of dietary energy, assuming an energy intake of 8400 kJ/day for an adult female, as per the rationale above [37].

e. The upper limit for Sodium was based on suggested dietary target to reduce chronic disease in the nutrient reference values (NRVs) (<2,000 mg/day sodium) [37].

Table S2. Reference Values from NRF 9.3 [36] and corresponding Australian Nutrient Reference Values [37] used in the current study.

	RDV (US)	MRV (US)	RDI/AI (AUS)	MRV (AUS)
Protein (g)	50		64	
Fibre (g)	25		30	
Vitamin A	5000 IU		900 μg/day	
Vitamin C (mg)	60		45	
Vitamin E (mg)	20		10	
Calcium (mg)	1000		1000	
Iron (mg)	18		18	
Potassium (mg)	3500		3800	
Magnesium (mg)	400		420	
Saturated fat (g)		20		20
Added Sugar (g)		50		50
Sodium (mg)		2400		2000

RDV: Reference daily value. MRV: Maximum recommended value. US: United States. RDI: Recommended dietary intake. AI: Adequate intake. AUS: Australia.

Supplementary Methods S2: Rules used to develop the substitution modelling protocol.

The substitution modelling algorithm was developed and employed according to the following substitution rules:

1. All substituted healthier diets aim to meet Australian Dietary Guidelines for core food groups [39], with no allowance for discretionary foods. To reduce complexity in the modelling protocol and align with consumer perceptions, legumes were considered in the meat and alternatives core food group only.

2. Number of servings for each core food group was calculated by dividing the weight of each food category consumed by the weight per serve, as defined in the Australian Dietary Guidelines [39], followed by summing the serves of all food categories within a core food group. For example, for the vegetables core food group, the number of servings was totalled from the following food categories: potatoes, unprocessed; orange/yellow vegetables; green leafy vegetables; other vegetables; vegetable dishes. One discretionary serving was calculated as being equivalent to 150 kcal.

3. All substitutions replaced food categories in the current diet placed in the lowest three nutrient density to cost ratio quartiles with core food categories from the highest quartile of nutrient density to cost, as identified in Step 1 of the modelling protocol.

4. Where more than one replacement option in the highest quartile was possible, selection of the replacement food category was random. For example, ready to eat breakfast cereals, unfortified, sugars >20g/100g, could

be replaced with ready to eat breakfast cereals, fortified, sugars ≤20g/100g; ready to eat breakfast cereals, unfortified, sugars ≤20g/100g; or hot porridge.

5. If no food category was appropriate to be substituted using the highest quartile of nutrient density to cost, the highest ranking food category from the second quartile was used.

6. Each substitution was made according to the 'like for like' principle, whereby each food category in the current diet was substituted for a healthier (more nutrient-dense) version of that food, keeping as close to the original food as possible. For example, white bread was substituted with wholegrain bread or processed meat was substituted with lean fresh meat.

7. The substitution of discretionary food categories for core food categories was also made according to the 'like for like' principle. For example, sweetened beverages were replaced with fruit juices. Where a direct substitution was not possible due to there being no similar core food, the selection of the replacement food was based upon cost and the judgment of the investigators. For example, chocolate bars were replaced with a random selection from the top quartile of food categories from the grains and cereals core food group.

8. If a 'like for like' substitution was not possible due to the dietary guidelines for that food category already being met; or the food category being discretionary and needing to be replaced with a core food category, the substitution was made using a more nutrient-dense, lower cost food category from the next most lacking core food group, according to the following hierarchy, based on the highest to the lowest level of intake within the current Australian diet across both children and adults [1]: Vegetables > Meat and alternatives > Dairy and dairy alternatives > Cereals and grain foods > Fruit.

9. In cases when a healthier version of the food to be substituted was not available, the selection of the replacement food was based upon cost and the judgment of the investigators.

Table S3. Nutrient density (NRF9.3/100 kcal), cost (AUD/100 kcal) and the nutrient density to cost ratio [1] (with quartile rankings) for all 57 food categories. The classification according to the NOVA processing levels is also shown.

Food categories	NRF9.3/100 kcal (SD)	AUD/100 kcal (SD)	Nutrient density to cost ratio Value (SD)	Quartile	NOVA processing classification
**Core food categories**					
**Vegetables**					
Potatoes, unprocessed	97.07 (4.6)	0.48 (0.1)	216.94 (73.2)	1	Unprocessed
Orange/yellow vegetables	302.23 (126.0)	0.69 (0.1)	492.29 (304.5)	1	Unprocessed
Other vegetables	443.02 (160.6)	2.16 (1.4)	293.39 (171.9)	1	Unprocessed
Green leafy vegetables	479.24 (123.0)	6.01 (3.6)	166.15 (191.0)	2	Unprocessed
Vegetable dishes	54.39 (39.8)	0.76 (0.2)	66.15 (36.6)	3	Unprocessed
**Fruit**					
Fruit juice	273.65 (124.8)	0.46 (0.2)	650.08 (318.9)	1	Processed
Dried fruit	28.56 (9.0)	0.19 (0.1)	170.99 (80.1)	1	Unprocessed
Whole fruit	55.74 (21.3)	0.74 (0.3)	92.31 (60.9)	2	Unprocessed
Commercial fruit, in syrup	50.94 (59.9)	0.61 (0.2)	87.38 (106.3)	2	Ultra-processed
Commercial fruit, in juice	50.97 (22.5)	0.86 (0.2)	61.84 (24.3)	3	Processed
**Cereals and grains**					

Rice, grains and flours	12.87 (4.6)	0.04 (0.0)	348.27 (196.5)	1	Unprocessed
Pasta and noodles, plain	20.71 (9.0)	0.07 (0.0)	304.58 (43.6)	1	Unprocessed
Hot porridge	22.61 (7.6)	0.15 (0.1)	290.93 (219.8)	1	Unprocessed
Breads, wholegrain and fortified	25.14 (3.1)	0.12 (0.0)	212.96 (57.7)	1	Ultra-processed
RTE breakfast cereals, fortified, sugar ≤20g/100g	25.25 (26.4)	0.12 (0.0)	200.07 (178.4)	1	Ultra-processed
RTE breakfast cereals, unfortified, sugar ≤20g/100g	24.90 (10.8)	0.18 (0.1)	176.09 (117.8)	1	Ultra-processed
Breads and rolls, refined grain, fortified	12.11 (1.7)	0.11 (0.0)	116.16 (15.9)	2	Ultra-processed
Breads and rolls, wholegrain (all grains), unfortified	29.19 (2.8)	0.30 (0.2)	130.19 (67.7)	2	Ultra-processed
RTE breakfast cereals, unfortified, sugar >20g/100g	14.19 (17.4)	0.31 (0.0)	52.37 (39.9)	3	Ultra-processed
Pasta and noodles, with additions	26.28 (21.7)	0.89 (0.3)	29.38 (23.2)	3	Ultra-processed
Breads and rolls, refined grain (white), unfortified	17.11 (2.5)	0.69 (0.4)	34.40 (19.0)	3	Ultra-processed
Batter-based products	−0.64 (4.7)	0.52 (0.2)	2.80 (2.3)	4	Ultra-processed
**Dairy and dairy alternatives**					
Dairy milk substitutes	50.07 (30.7)	0.27 (0.1)	214.06 (161.3)	1	Ultra-processed
Dairy milk, reduced fat or skim	58.00 (13.0)	0.30 (0.1)	209.92 (79.0)	1	Unprocessed
Dairy milk, full fat	24.29 (1.8)	0.17 (0.0)	146.43 (45.7)	2	Unprocessed
Liquid breakfast, fortified beverages	77.03 (4.6)	0.85 (0.1)	91.46 (10.1)	2	Ultra-processed
Yoghurt, reduced fat	32.39 (19.9)	0.48 (0.2)	64.54 (21.0)	3	Ultra-processed
Flavoured milk	27.61 (20.4)	0.48 (0.3)	56.61 (13.6)	3	Ultra-processed
Cheese	6.25 (5.5)	0.23 (0.0)	26.13 (20.1)	4	Processed
Ultra-processed cheese	−1.99 (9.7)	0.31 (0.1)	10.15 (23.7)	4	Ultra-processed
Yoghurt, full fat	12.14 (13.3)	0.43 (0.1)	23.58 (18.3)	4	Unprocessed
**Meat and alternatives**					
Legumes and legume dishes	42.56 (5.6)	0.19 (0.0)	231.38 (50.0)	1	Processed
Eggs, without additions	42.41 (0.0)	0.48 (0.1)	90.96 (16.0)	2	Unprocessed
Meat substitutes and dishes	66.92 (16.5)	0.80 (0.4)	113.09 (63.4)	2	Ultra-processed
Seeds and nuts	4.73 (33.3)	0.20 (0.2)	150.56 (135.5)	2	Unprocessed
Fish, unprocessed, without additions	60.47 (7.2)	1.29 (0.3)	50.22 (18.0)	3	Unprocessed
Fish, processed, tinned	32.13 (21.7)	0.50 (0.3)	84.88 (70.8)	3	Processed
Red meat, fat fully-trimmed	35.93 (8.0)	1.36 (0.3)	26.74 (4.4)	3	Unprocessed
Red meat, untrimmed or semi-trimmed	20.09 (8.7)	0.68 (0.2)	32.52 (19.2)	3	Unprocessed
Poultry	26.19 (14.9)	0.32 (0.1)	79.67 (38.5)	3	Unprocessed
**Other foods**					
Margarines and margarine-like spreads	10.19 (3.6)	0.08 (0.0)	135.42 (47.8)	2	Ultra-processed
Oils	5.84 (1.0)	0.04 (0.0)	154.91 (26.6)	2	Culinary processed
**Discretionary food categories**					
Potatoes, processed	37.34 (4.9)	0.10 (0.0)	403.54 (109.3)	1	Ultra-processed
RTE breakfast cereals, fortified, sugar >20g/100g	23.62 (22.4)	0.17 (0.0)	136.83 (107.0)	2	Ultra-processed
Dips	23.73 (22.9)	0.38 (0.2)	87.44 (71.6)	2	Ultra-processed
Savoury snacks	8.91 (15.0)	0.19 (0.0)	59.77 (72.9)	3	Ultra-processed

Sweet snacks	14.07 (20.0)	0.25 (0.1)	69.93 (65.0)	3	Ultra-processed
Muffins and scones	−0.26 (5.1)	0.38 (0.2)	5.60 (5.9)	4	Ultra-processed
Ultra-processed meats	−9.58 (0.6)	0.23 (0.0)	0.00 (0.0)	4	Ultra-processed
Processed meat	−22.29 (8.4)	0.74 (0.4)	0.00 (0.0)	4	Processed
Cream	−10.13 (4.6)	0.21 (0.1)	0.00 (0.0)	4	Culinary processed
Sweet spreads and syrups	−14.29 (15.1)	0.19 (0.1)	1.19 (1.8)	4	Ultra-processed
Savoury biscuits	−0.19 (1.5)	0.12 (0.0)	4.30 (5.2)	4	Ultra-processed
Fish processed, battered and crumbed	6.26 (3.0)	0.37 (0.1)	18.06 (11.0)	4	Ultra-processed
Solid fats	−0.02 (2.8)	0.16 (0.0)	7.58 (8.2)	4	Culinary processed
Sweetened beverages	−13.45 (43.5)	5.88 (12.9)	0.73 (0.8)	4	Ultra-processed
Desserts	−14.26 (17.7)	0.44 (0.7)	0.00 (0.0)	4	Ultra-processed

[1] The nutrient density to cost ratio was calculated according to the following formula: (mean NRF9.3/100 kcal) / (mean cost/100 kcal).

Table S4. Micronutrient analysis [1] of the **(a)** current and **(b)** healthier, low-cost diets for each member of both low and medium socioeconomic households.

(a)

Current diet - Micronutrients (per day)	Low socioeconomic household					Middle socioeconomic household				
	Male 31–50 y	Female 31–50 y	Male 9–13 y	Female 4–8 y	Total	Male 31–50 y	Female 31–50 y	Male 9–13 y	Female 4–8 y	Total
n	281	301	73	74		324	408	86	73	
					**Current Diet**					
Vitamin A (ug)	571.3 (86.1)	526.4 (33.4)	461.8 (41.3)	493.1 (40.6)	2052.6 (109.0)	504.2 (39.8)	572.5 (35.5)	497.6 (51.6)	483.0 (27.1)	2057.4 (79.0)
Thiamine (mg)	1.4 (0.1)	1.3 (0.1)	1.2 (0.1)	0.9 (0.0)	4.8 (0.2)	1.6 (0.1)	1.4 (0.0)	1.1 (0.1)	1.1 (0.0)	5.0 (0.1)
Riboflavin (mg)	2.1 (0.2)	1.8 (0.1)	1.9 (0.1)	1.4 (0.1)	7.2 (0.3)	2.3 (0.2)	1.9 (0.1)	1.9 (0.1)	1.5 (0.1)	7.6 (0.3)
Niacin equivalents (mg)	25.4 (1.5)	28.0 (1.1)	21.6 (1.2)	19.9 (0.7)	94.9 (2.3)	27.0 (1.3)	28.6 (0.9)	19.3 (1.1)	22.9 (0.7)	97.8 (2.0)
Vitamin B6 (ug)	1.1 (0.1)	1.1 (0.0)	0.9 (0.0)	0.8 (0.0)	3.8 (0.1)	1.1 (0.1)	1.1 (0.0)	0.9 (0.1)	0.9 (0.0)	4.0 (0.1)
Folate equivalents (ug)	492.5 (28.8)	483.5 (19.8)	408.0 (22.0)	363.6 (13.7)	1746.4 (43.5)	584.2 (31.2)	490.2 (15.8)	391.2 (23.5)	389.3 (11.8)	1854.9 (43.8)
Vitamin B12 (ug)	4.2 (0.5)	4.1 (0.2)	3.7 (0.3)	3.0 (0.1)	15.0 (0.6)	4.3 (0.4)	4.2 (0.2)	3.5 (0.3)	3.5 (0.2)	15.5 (0.6)
Vitamin E (ug)	6.1 (0.5)	6.2 (0.3)	5.5 (0.3)	4.9 (0.2)	22.7 (0.7)	6.7 (0.4)	6.2 (0.2)	5.2 (0.3)	5.8 (0.2)	23.9 (0.6)
Calcium(mg)	974.3 (102.4)	811.7 (38.7)	879.0 (69.2)	670.2 (29.6)	3335.3 (132.8)	989.2 (87.4)	814.4 (35.3)	898.5 (73.8)	708.9 (27.6)	3411.4 (122.9)
Iron (mg)	7.6 (0.4)	7.9 (0.3)	6.4 (0.4)	5.4 (0.2)	27.4 (0.7)	9.1 (0.5)	8.3 (0.3)	6.1 (0.3)	6.4 (0.2)	29.9 (0.7)
Magnesium (mg)	239.7 (15.8)	245.5 (9.4)	207.5 (10.2)	181.9 (6.1)	874.6 (21.9)	255.9 (12.9)	247.3 (7.6)	205.2 (11.8)	205.0 (5.6)	913.4 (19.9)
Potassium (mg)	2521.3 (186.2)	2389.4 (85.2)	2081.1 (111.9)	1855.8 (65.2)	8847.4 (242.3)	2465.2 (143.7)	2461.9 (77.6)	2167.6 (125.7)	2064.2 (58.3)	9158.9 (214.2)
Sodium (mg)	1737.4 (112.5)	1665.3 (66.9)	1439.6 (84.3)	1181.1 (46.5)	6023.4 (162.5)	1795.0 (111.2)	1722.5 (64.1)	1401.8 (72.1)	1328.2 (42.5)	6247.5 (153.2)
Zinc (mg)	7.3 (0.5)	7.8 (0.3)	6.3 (0.3)	5.6 (0.2)	27.0 (0.7)	7.7 (0.4)	8.1 (0.3)	5.8 (0.3)	6.3 (0.2)	27.9 (0.6)

(b)

Modelled healthier diet - Micronutrients (per day)	Low socioeconomic household					Middle socioeconomic household					Statistical comparison (*p*-value) [2]	
	Male 31–50 y	Female 31–50 y	Male 9–13 y	Female 4–8 y	Total	Male 31–50 y	Female 31–50 y	Male 9–13 y	Female 4–8 y	Total	Diet low SES	Diet med SES
n	281	301	73	74		324	408	86	73			
Vitamin A (ug)	1433.6 (170.6)	1355.7 (109.7)	981.3 (119.5)	1114.1 (79.4)	4884.7 (248.4)	1444.0 (168.3)	1421.7 (89.0)	1029.7 (134.5)	1086.9 (57.4)	4982.3 (240.1)	<0.001	<0.001
Thiamine (mg)	1.4 (0.1)	1.5 (0.1)	1.2 (0.1)	1.0 (0.0)	5.1 (0.2)	1.6 (0.1)	1.5 (0.1)	1.1 (0.1)	1.1 (0.0)	5.3 (0.2)	0.001	<0.001
Riboflavin (mg)	2.4 (0.2)	2.3 (0.1)	1.8 (0.1)	1.7 (0.1)	8.2 (0.3)	2.6 (0.2)	2.2 (0.1)	1.9 (0.2)	1.8 (0.1)	8.5 (0.3)	<0.001	<0.001
Niacin equivalents (mg)	25.3 (1.5)	27.6 (1.1)	20.0 (1.0)	19.2 (0.7)	92.2 (2.2)	27.6 (1.4)	26.7 (0.8)	20.3 (1.4)	21.0 (0.6)	95.6 (2.2)	0.173	0.252

Vitamin B6 (ug)	1.2 (0.1)	1.3 (0.0)	0.9 (0.0)	0.9 (0.0)	4.4 (0.1)	1.3 (0.1)	1.3 (0.0)	1.0 (0.1)	1.0 (0.0)	4.6 (0.1)	<0.001	<0.001
Folate equivalents (ug)	660.4 (36.5)	707.5 (27.3)	543.8 (29.7)	506.5 (19.0)	2418.2 (57.6)	735.1 (35.7)	725.6 (23.8)	538.2 (31.0)	534.7 (15.4)	2533.6 (55.1)	<0.001	<0.001
Vitamin B12 (ug)	4.4 (0.5)	4.2 (0.3)	3.2 (0.3)	3.0 (0.2)	14.8 (0.7)	4.5 (0.6)	3.8 (0.2)	3.6 (0.4)	3.3 (0.2)	15.2 (0.8)	0.750	0.631
Vitamin E (ug)	7.2 (0.6)	8.1 (0.4)	6.6 (0.5)	5.9 (0.3)	27.7 (0.9)	7.7 (0.5)	8.1 (0.3)	6.1 (0.4)	6.6 (0.2)	28.6 (0.7)	<0.001	<0.001
Cal-cium(mg)	1326.1 (135.6)	1315.9 (86.8)	938.4 (77.8)	928.1 (47.9)	4508.4 (185.1)	1370.5 (150.4)	1208.1 (64.0)	1062.3 (109.2)	1009.0 (53.2)	4649.8 (203.6)	<0.001	<0.001
Iron (mg)	11.0 (0.7)	12.3 (0.5)	9.3 (0.6)	8.6 (0.3)	41.1 (1.1)	12.2 (0.6)	12.8 (0.5)	9.2 (0.5)	9.8 (0.3)	44.0 (1.0)	<0.001	<0.001
Magnesium (mg)	358.9 (22.0)	390.8 (14.7)	291.4 (15.0)	278.4 (9.8)	1319.6 (32.0)	387.1 (19.0)	386.6 (12.4)	302.1 (18.1)	308.4 (9.2)	1384.2 (30.4)	<0.001	<0.001
Potassium (mg)	3955.8 (245.6)	4148.7 (157.4)	2997.5 (146.0)	3030.5 (102.6)	14132.5 (342.0)	4253.6 (240.8)	4080.9 (130.4)	3323.6 (195.2)	3327.9 (101.1)	14986.0 (342.1)	<0.001	<0.001
Sodium (mg)	1621.1 (118.9)	1863.7 (87.9)	1256.8 (87.3)	1277.8 (56.2)	6019.4 (180.7)	1695.6 (97.1)	1894.5 (76.6)	1320.3 (101.0)	1445.3 (49.4)	6355.7 (167.1)	0.983	0.541
Zinc (mg)	8.2 (0.6)	9.0 (0.4)	6.2 (0.4)	6.3 (0.2)	29.8 (0.8)	8.8 (0.5)	8.8 (0.3)	6.6 (0.5)	6.9 (0.2)	31.2 (0.8)	<0.001	<0.001

[1] All data is mean (SEM). [2] Statistical comparison is between low and medium socioeconomic total households. Statistical comparison is between current vs healthier diet for low socioeconomic household (diet low SES); or current vs healthier diet for medium socioeconomic household (diet med SES). *p*-values comparing diets are obtained from a general linear model for change in values with SES, age-sex group and their interaction as fixed factors. For statistical significance, $p < 0.005$. SES, socioeconomic status.

Table S5. Individual component scores for the HEIFA-2013 and DGI-CA for each member of both low and medium SES households.

	Low SES household			Medium SES household		
	**Current diet**	**Healthier diet**	***p*-value [2]**	**Current diet**	**Healthier diet**	***p*-value [2]**
			**HEIFA-2013 Scores [1]**			
**Male (31–50 years)**						
n	281	281		324	324	
Component 1: Discretionary	5.0 (0.3)	10.0 (0.0)	<0.001	4.8 (0.2)	10.0 (0.0)	<0.001
Component 2: Vegetables	1.7 (0.1)	6.0 (0.1)	<0.001	1.7 (0.1)	6.5 (0.1)	<0.001
Component 3: Fruits	2.0 (0.2)	2.2 (0.2)	0.500	2.3 (0.2)	2.8 (0.2)	0.076
Component 4: Grains	2.8 (0.2)	4.5 (0.2)	<0.001	3.1 (0.2)	4.7 (0.2)	<0.001
Component 5: Meat	2.7 (0.2)	4.4 (0.3)	<0.001	2.9 (0.2)	4.7 (0.2)	<0.001
Component 6: Dairy	3.8 (0.2)	4.7 (0.3)	0.008	3.7 (0.2)	4.7 (0.2)	0.001
Component 7: Water	5.0 (0.0)	5.0 (0.0)	-	5.0 (0.0)	5.0 (0.0)	-
Component 8: Fat	2.0 (0.1)	5.3 (0.1)	<0.001	1.8 (0.1)	5.2 (0.1)	<0.001
Component 9: Sodium	5.1 (0.3)	10.0 (0.0)	<0.001	4.7 (0.3)	9.8 (0.1)	<0.001
Component 10: Added sugars	8.7 (0.2)	10.0 (0.0)	<0.001	8.7 (0.2)	10.0 (0.0)	<0.001
Component 11: Alcohol	5.0 (0.0)	5.0 (0.0)	-	5.0 (0.0)	5.0 (0.0)	-
HEIFA-2013 TO-TAL SCORE	43.8 (0.7)	67.1 (0.6)	<0.001	43.7 (0.6)	68.5 (0.6)	<0.001

**Female (31–50 years)**						
n	301	301		408	408	
Component 1: Discretionary	6.0 (0.2)	10.0 (0.0)	<0.001	5.6 (0.2)	10.0 (0.0)	<0.001
Component 2: Vegetables	1.5 (0.1)	5.6 (0.1)	<0.001	1.7 (0.1)	6.2 (0.1)	<0.001
Component 3: Fruits	2.1 (0.2)	2.6 (0.2)	0.076	2.5 (0.1)	2.7 (0.2)	0.259
Component 4: Grains	2.2 (0.1)	3.4 (0.2)	<0.001	2.2 (0.1)	3.3 (0.1)	<0.001
Component 5: Meat	2.6 (0.2)	3.9 (0.2)	<0.001	3.2 (0.2)	4.9 (0.2)	<0.001
Component 6: Dairy	3.3 (0.2)	3.8 (0.2)	0.080	3.3 (0.2)	4.2 (0.2)	<0.001
Component 7: Water	5.0 (0.0)	5.0 (0.0)	-	5.0 (0.0)	5.0 (0.0)	-
Component 8: Fat	1.9 (0.1)	5.5 (0.1)	<0.001	1.8 (0.1)	5.6 (0.1)	<0.001
Component 9: Sodium	5.7 (0.3)	10.0 (0.0)	<0.001	5.2 (0.2)	10.0 (0.0)	<0.001
Component 10: Added sugars	8.6 (0.2)	10.0 (0.0)	<0.001	9.2 (0.1)	10.0 (0.0)	<0.001
Component 11: Alcohol	5.0 (0.0)	5.0 (0.0)	-	5.0 (0.0)	5.0 (0.0)	-
HEIFA-2013 TOTAL SCORE	44.0 (0.6)	64.9 (0.6)	<0.001	44.6 (0.5)	66.8 (0.5)	<0.001
			**DGI-CA Scores** [1]			
**Male (9-13 years)**						
n	73	73		86	86	
Component 1: Variety	6.1 (0.2)	7.8 (0.2)	<0.001	6.1 (0.2)	8.1 (0.2)	<0.001
Component 2: Vegetables	2.2 (0.4)	8.7 (0.3)	<0.001	1.6 (0.3)	9.2 (0.2)	<0.001
Component 3: Fruits	5.9 (0.5)	6.2 (0.5)	0.667	5.9 (0.5)	6.2 (0.5)	0.677
Component 4: Grains	2.8 (0.3)	6.3 (0.4)	<0.001	3.6 (0.3)	6.7 (0.3)	<0.001
Component 5: Meat	3.3 (0.5)	5.7 (0.6)	0.003	4.0 (0.5)	6.4 (0.5)	<0.001
Component 6: Dairy	3.3 (0.3)	5.4 (0.4)	<0.001	3.0 (0.3)	5.2 (0.4)	<0.001
Component 7: Water	10.0 (0.0)	10.0 (0.0)	-	10.0 (0.0)	10.0 (0.0)	-
Component 8: Discretionary/extra foods	5.1 (0.9)	20.0 (0.0)	<0.001	3.7 (0.7)	20.0 (0.0)	<0.001
Component 9: Saturated fat	5.4 (0.1)	6.7 (0.2)	<0.001	5.4 (0.1)	7.2 (0.1)	<0.001
DGI-CA TOTAL SCORE	44.0 (1.8)	76.8 (1.4)	<0.001	43.2 (1.5)	79.1 (1.3)	<0.001

**Female (4-8 years)**						
n	74	74		73	73	
Component 1: Variety	6.2 (0.2)	7.9 (0.2)	<0.001	6.4 (0.2)	8.1 (0.2)	<0.001
Component 2: Vegetables	1.8 (0.3)	8.9 (0.3)	<0.001	2.5 (0.4)	9.4 (0.2)	<0.001
Component 3: Fruits	6.9 (0.5)	7.2 (0.5)	0.691	7.9 (0.4)	7.9 (0.4)	1
Component 4: Grains	3.7 (0.3)	6.9 (0.3)	<0.001	3.7 (0.3)	6.7 (0.3)	<0.001
Component 5: Meat	3.8 (0.5)	6.0 (0.5)	0.004	3.1 (0.5)	5.8 (0.6)	<0.001
Component 6: Dairy	3.9 (0.3)	5.4 (0.3)	<0.001	3.8 (0.3)	5.3 (0.4)	0.002
Component 7: Water	10.0 (0.0)	10.0 (0.0)	-	10.0 (0.0)	10.0 (0.0)	-
Component 8: Discretion-ary/extra foods	7.4 (1.0)	20.0 (0.0)	<0.001	6.9 (1.0)	20.0 (0.0)	<0.001
Component 9: Saturated fat	5.4 (0.1)	7.3 (0.1)	<0.001	5.2 (0.1)	7.5 (0.1)	<0.001
DGI-CA TOTAL SCORE	49.0 (1.6)	79.5 (1.2)	<0.001	49.5 (1.7)	80.6 (1.1)	<0.001

[1] All scores are mean (SEM). [2] Statistical significance is set at $p < 0.005$. A hyphen indicates that the values for comparison were exactly the same among all samples from within a group, and thus between both current vs healthier diets, and low vs middle socioeconomic households.
